# Non-random aneuploidy specifies subgroups of pilocytic astrocytoma and correlates with older age

**DOI:** 10.18632/oncotarget.5571

**Published:** 2015-09-10

**Authors:** Adam M. Fontebasso, Margret Shirinian, Dong-Anh Khuong-Quang, Denise Bechet, Tenzin Gayden, Marcel Kool, Nicolas De Jay, Karine Jacob, Noha Gerges, Barbara Hutter, Huriye Şeker-Cin, Hendrik Witt, Alexandre Montpetit, Sébastien Brunet, Pierre Lepage, Geneviève Bourret, Almos Klekner, László Bognár, Peter Hauser, Miklós Garami, Jean-Pierre Farmer, Jose-Luis Montes, Jeffrey Atkinson, Sally Lambert, Tony Kwan, Andrey Korshunov, Uri Tabori, V. Peter Collins, Steffen Albrecht, Damien Faury, Stefan M. Pfister, Werner Paulus, Martin Hasselblatt, David T.W. Jones, Nada Jabado

**Affiliations:** ^1^ Division of Experimental Medicine, McGill University and McGill University Health Centre, Montreal, Quebec, Canada; ^2^ Department of Experimental Pathology, Immunology and Microbiology, American University Of Beirut, Beirut, Lebanon; ^3^ Departments of Pediatrics and Human Genetics, McGill University and McGill University Health Centre, Montreal, Quebec, Canada; ^4^ Division of Pediatric Neurooncology, German Cancer Research Centre (DKFZ), Heidelberg, Germany; ^5^ Division of Theoretical Bioinformatics, German Cancer Research Centre (DKFZ), Heidelberg, Germany; ^6^ Department of Pediatric Oncology, Hematology and Immunology, University of Heidelberg, Heidelberg, Germany; ^7^ McGill University and Genome Quebec Innovation Centre, Montreal, Quebec, Canada; ^8^ Department of Neurosurgery, Medical and Health Science Center, University of Debrecen, Debrecen, Hungary; ^9^ 2nd Department of Paediatrics, Semmelweis University, Budapest, Hungary; ^10^ Department of Neurosurgery, Montreal Children's Hospital and McGill University Health Centre, Montreal, Canada; ^11^ Division of Molecular Histopathology, Department of Pathology, University of Cambridge, Cambridge, United Kingdom; ^12^ Clinical Cooperation Unit Neuropathology, German Cancer Research Center (DKFZ), Heidelberg, Germany; ^13^ Division of Pediatric Hematology-Oncology and The Labatt Brain Tumour Research Centre, The Hospital for Sick Children, Toronto, Canada; ^14^ Department of Pathology, Montreal Children's Hospital and McGill University Health Centre, Montreal, Canada; ^15^ Institute of Neuropathology, University Hospital Münster, Münster, Germany

**Keywords:** pilocytic astrocytoma, aneuploidy, BRAF, MDM2, PLK2, aging

## Abstract

Pilocytic astrocytoma (PA) is the most common brain tumor in children but is rare in adults, and hence poorly studied in this age group. We investigated 222 PA and report increased aneuploidy in older patients. Aneuploid genomes were identified in 45% of adult compared with 17% of pediatric PA. Gains were non-random, favoring chromosomes 5, 7, 6 and 11 in order of frequency, and preferentially affecting non-cerebellar PA and tumors with *BRAF* V600E mutations and not with *KIAA1549-BRAF* fusions or *FGFR1* mutations. Aneuploid PA differentially expressed genes involved in CNS development, the unfolded protein response, and regulators of genomic stability and the cell cycle (*MDM2*, *PLK2*),whose correlated programs were overexpressed specifically in aneuploid PA compared to other glial tumors. Thus, convergence of pathways affecting the cell cycle and genomic stability may favor aneuploidy in PA, possibly representing an additional molecular driver in older patients with this brain tumor.

## INTRODUCTION

Pilocytic astrocytoma (PA) is a World Health Organization (WHO) grade I astrocytoma and the most common pediatric brain tumor [1, 2]. These tumors generally have an excellent prognosis and limited morbidity when amenable to gross total surgical resection [2, 3], with the highest age-adjusted incidence rate occurring in children between 0-19 years old when assessed across the lifespan [4]. In children, PAs most commonly occur in the cerebellum, and with decreasing frequency in other more surgically challenging brain locations including the optic pathway, thalamus, brainstem, spinal cord and cerebral hemispheres [5]. In adults, the reverse gradient for location is observed, and the low incidence of PA largely contributes to the relative lack of molecular investigations in this setting [5].

Recent genomic studies have shown that, in children, virtually all PAs can be categorized as harboring an alteration leading to constitutive activation of the MAPK pathway. The most common genetic alteration (~65%) is a tandem duplication at 7q34 creating an in-frame fusion gene between *BRAF* and *KIAA1549* [6–9]. Other alterations of the pathway include *RAF*-family fusions [7, 10], *BRAF* V600E mutation [11], germline *NF1* alterations (reviewed in [8]), and fibroblast growth factor receptor 1 (*FGFR1*) and NTRK-family alterations [9, 12]. In contrast, lower frequencies of *RAF* fusion transcripts have been reported in adult PAs [13]. Importantly, 50-60% of PA in patients over the age of 20 years lacked a KIAA1549:BRAF fusion, BRAF V600E mutation or FGFR1 mutation [14]. This suggests that these tumors may harbor some of the rarer MAPK pathway alterations, or that other putative driver alterations or programs may additionally contribute to the molecular landscape of adult PA.

Whole-chromosomal copy number gains have been reported in a small cohort of 44 PA analyzed using array-based comparative genomic hybridization (aCGH) and seemed to favor older patients [15]. The prevalence, relation to patient age and significance of this phenotype as well as its relation to *BRAF* and MAPK alterations and possible underlying mechanisms remain, however, largely uncharacterized. As such, we analyzed a large combined dataset of 222 adult and pediatric PAs with whole-chromosomal copy number data, including tumors from previously published aCGH and SNP array studies [6, 7, 15–17]. We show that aneuploidy is a common feature of the genomes of adult PA, affecting 45% of tumors in this age range. This is a non-random process that favors gains but not losses, specifically in chromosomes 5, 7, 6 and 11. It is strongly associated to non-cerebellar regions, which are less amenable to gross-total surgical resection, and to tumors harboring *BRAF* V600E mutation, which may be one of the molecular drivers of this phenotype. Pathways favoring aneuploidy are differentially regulated in adult PA, with many expression changes mapping to chromosome 5. This includes central nervous system (CNS) developmental pathways, unfolded protein response pathways and the cell cycle, including overexpression of *MDM2* and *PLK2*, and specific gene expression signatures we identify to be strongly correlated with aneuploid PA. Furthermore, our findings suggest a link between aging and the physiological non-random aneuploidy which occurs in the brain and may predispose to this brain tumor.

## RESULTS

### Non-random aneuploidy characterizes adult PA tumors arising throughout the CNS

**Figure 1:**
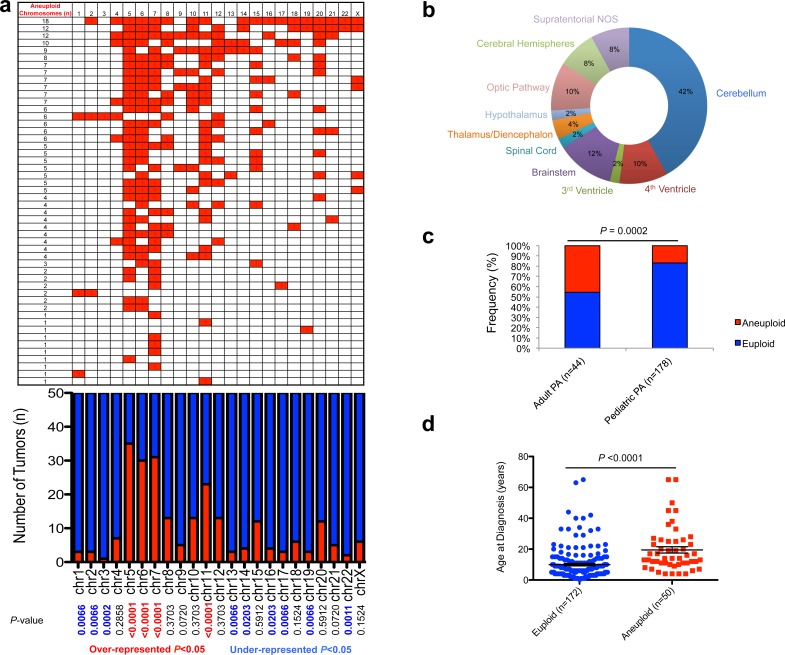
Characterization of aneuploidy observed in pilocytic astrocytoma **a.**, Gains in 50 aneuploid PA samples (upper panel), frequency of specific gains, and significance (*P* < 0.05; Fisher's Exact Test; lower panel). **b.**, Neuroanatomical distribution of aneuploid PA tumors favors non-cerebellar areas. **c.**, Increased aneuploidy observed amongst adult PA tumors (*P* = 0.0002; Fisher's Exact Test). **d.**, Age of patients with aneuploid and euploid PA tumors (*P* < 0.0076; unpaired two-tailed *t*-test).

We combined published datasets of whole-chromosomal copy number analyses of PA tumors [6, 7, 15–17] with newly-derived profiles from DNA methylation data [10] as well as newly-analyzed tumor samples, and assembled copy number data for a combined series of 222 PAs (*n* = 44 adult; *n* = 178 pediatric). In our combined dataset, whole chromosomal gain in at least one chromosome was common, occurring in 22.5% (50/222) tumors overall (Figure 1a–1d; Supplementary Table 1). This aneuploidy was present in PA tumors occurring throughout the central nervous system (CNS), with a notable fraction occurring in surgically challenging areas such as the thalamus, hypothalamus, optic pathway, brainstem, 4th ventricle and spinal cord (Figure 1b). Aneuploidy tended to favor tumors occurring in non-cerebellar areas when compared with the location distribution of the euploid cohort, a trend which approached statistical significance (*P* = 0.0552; Fisher's Exact Test). Aneuploidy was significantly more common in the adult PA subset (20/44, 45% vs. 30/178, 17%; P = 0.0002; Fisher's Exact Test) (Figure 1c) with the average age at diagnosis of patients with aneuploid PA tumors being about 10 years older than those with euploid tumors (19.5+/−2.1 years, compared to 9.9+/−0.8 years, respectively; *P* < 0.0001; unpaired, two-tailed t-test) (Figure 1d). The frequency of individual chromosomes affected demonstrated a specific pattern across the 50 aneuploid PAs, with the most commonly altered chromosomes in order of frequency being chromosomes 5, 7, 6 and 11, in keeping with previous findings [15]. These four chromosomes were statistically over-represented in overall events among the aneuploid tumors (*P* < 0.0001). In addition, chromosomes 1, 2, 3, 13, 14 16, 17, 19 and 22 were significantly under-represented across the background of all changes (*P* < 0.05) (Figure 1a). Aneuploidy did not appear to correlate with different progression-free survival (PFS) profiles through Kaplan-Meier analysis (Supplementary Figure 3).

### Aneuploidy and MAPK alterations in PA

**Figure 2:**
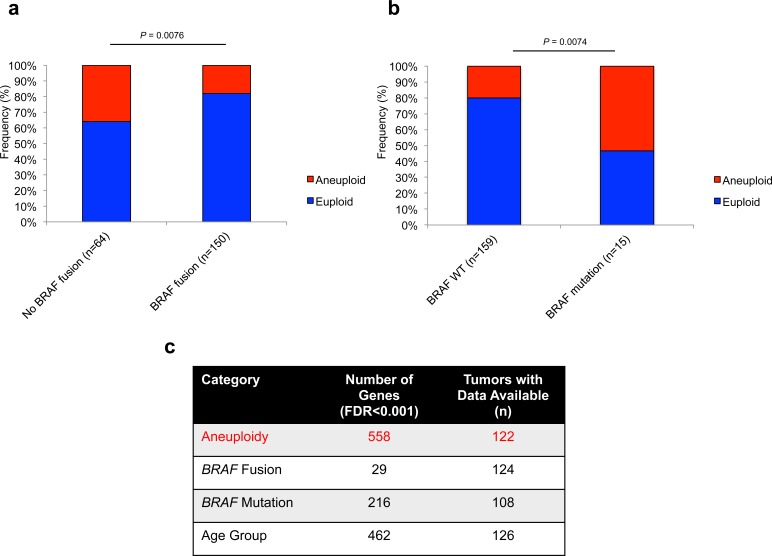
Aneuploidy is differentially associated with prominent MAPK alterations in PA with a strong effect on global gene expression **a.**, Frequency of aneuploidy within *BRAF* fusion (*P* = 0.0076; Fisher's Exact Test) **a.** and *BRAF* mutation (*P* = 0.0074; Fisher's Exact Test) **b.** subsets of PA tumors. **c.**, Strong effect of aneuploidy on global gene expression in PA at FDR < 0.001.

By integrating aneuploidy and BRAF fusion status, we determined that tumors wild-type for *BRAF* fusion demonstrated an increased rate of aneuploidy (36%, 23/64 vs 18%, 27/150; *P* = 0.0076; Fisher's Exact Test) (Figure 2a). Conversely, an increased frequency of aneuploidy is seen in tumors harboring BRAF mutations (53%, 8/15 vs 20%, 32/127; *P* = 0.0074; Fisher's exact test) (Figure 2b). Aneuploidy did not correlate significantly with FGFR1 mutation status (*P* = 0.68; Fisher's Exact Test). Using the R2 microarray analysis and visualization platform (http://r2.amc.nl), and identified differential expression of 558 genes (FDR < 0.001, ANOVA) between euploid (*n* = 93) and aneuploid tumors (*n* = 29) (Figure 2c). Other comparisons demonstrated fewer genes differentially regulated at FDR < 0.001, including BRAF fusion positive vs negative (29 genes), BRAF mutation positive vs negative (216 genes) and age group categories (462 genes), demonstrating a strong effect of aneuploidy on global gene expression patterns in PA (Figure 2c).

### Pathways aberrantly regulated in aneuploid tumors

**Figure 3:**
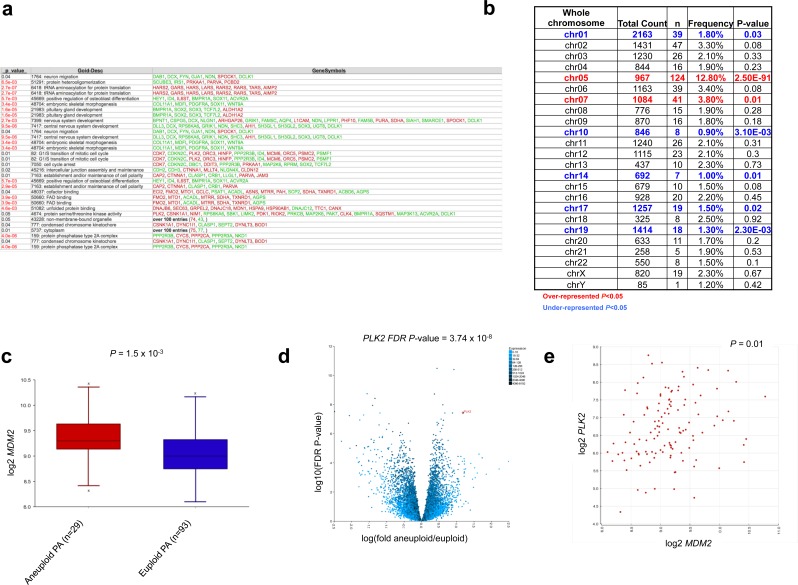
Comparative gene expression analysis identifies specific pathways aberrantly regulated in aneuploid tumors **a.**, Aneuploidy signatures demonstrate enrichment of CNS development, cell cycle and unfolded protein binding pathways. **b.**, Chromosomal mapping of the 558 genes demonstrates enrichment within chromosomes involved in aneuploid gains. **c.**, **d.**, Over-expression of candidates *MDM2* **c.** and *PLK2* (d) within the aneuploid PA subgroup (*P* = 1.5×10^−3^ and FDR adjusted *P*-value = 3.74×10^−8^ respectively; ANOVA). **e.**, Correlation of *MDM2* and *PLK2* expression in 122 PAs with available aneuploid data (*P* = 0.01).

Gene Ontology (GO) analysis of the 558 genes differentially regulated between euploid and aneuploid tumors revealed significance of molecular pathways including GO terms such as CNS development (GO: 7417), cell cycle arrest (GO: 7050), G1/S transition of the mitotic cell cycle (GO: 82) and unfolded protein binding (GO: 51082) (Figure 3a). Mapping of these 558 genes to their respective chromosomal locations revealed enrichment (over-representation of differentially expressed genes) on chromosomes 5 and 7, and under-representation of chromosomes 1, 10, 14, 17 and 19 (Figure 3b), mirroring the relative frequency of gains of these chromosomes seen in our cohort. Candidate genes significantly de-regulated between aneuploid and euploid PA tumors included *MDM2* (Figure 3c), encoding the E3 ubiquitin ligase MDM2, and *polo-like kinase 2* (*PLK2*) (Figure 3d). These genes were also strongly correlated with each other in the 122 PA tumors with available gene expression and ploidy status (*P* = 0.01, Figure 3e).

### *MDM2* is highly expressed in aneuploid PA

**Figure 4:**
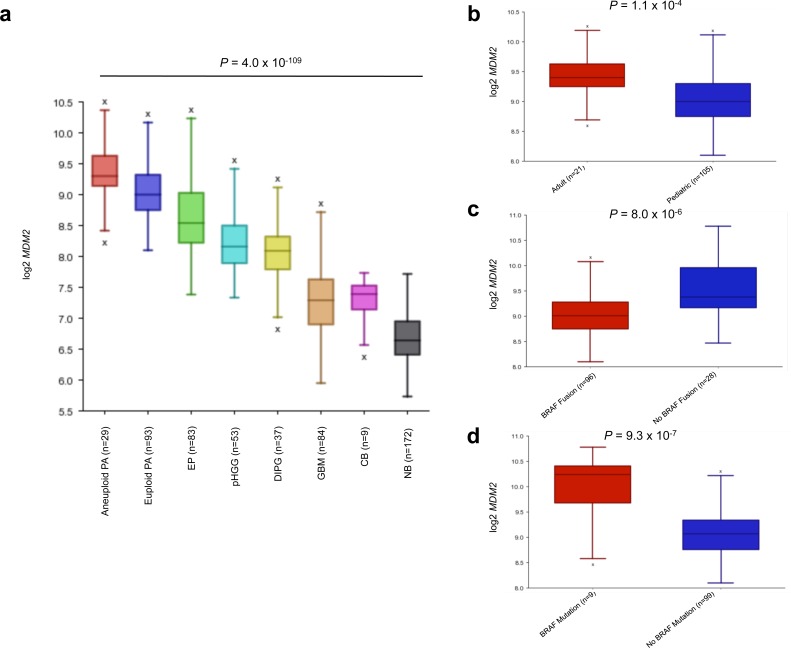
*MDM2* expression analysis of CNS tumors and tissues reveals high expression in aneuploid PA **a.**, *MDM2* expression is highest in aneuploid PA tumors (*P* = 4.0×10^−109^, ANOVA). NB = normal brain, CB = normal cerebellum, GBM = glioblastoma, DIPG = diffuse intrinsic pontine glioma, pHGG = pediatric high-grade glioma, EP = ependymoma, PA = pilocytic astrocytoma. **b.**, **c.**, **d.**, *MDM2* over-expression in adult patients (>18 years) (*P* = 1.1×10^−4^) **b.**, *BRAF* fusion negative (*P* = 8.0×10^−6^) **c.** and *BRAF* mutant PA (*P* = 9.3×10^−7^) **d.**. *P*-values calculated using ANOVA.

Utilizing the R2 database tool, we sought to investigate *MDM2* expression comparatively across PA, as well as in other gliomas and control brain samples. Across multiple gene expression datasets including PA, adult glioblastoma (GBM), pediatric high-grade glioma (pHGG), diffuse intrinsic pontine glioma (DIPG), ependymoma (EP) and normal brain (NB) and cerebellum (CB) samples, aneuploid PA tumors demonstrated the highest levels of *MDM2* expression (Figure 4a). Further dissection of *MDM2* expression within the PA dataset with available clinical and molecular variables revealed a strong association of *MDM2* over-expression in adult PA samples (*P* = 1.1×10^−4^; ANOVA) (Figure 4b), tumors wild-type for *BRAF* fusion (*P* = 8.0×10^−6^ ANOVA) (Figure 4c) and tumors harboring *BRAF* mutations (*P* = 9.3×10^−7^; ANOVA) (Figure 4d), mirroring our findings as to the association of these factors with aneuploidy. Even slight elevations in *MDM2* expression have been reported to accelerate tumorigenesis (e.g. in association with the *MDM2* SNP 309 [18, 19]). Manual curation of DNA copy number profiles revealed PA genomes largely deficient in focal copy number alterations other than *BRAF* duplication and whole-chromosomal aneuploidy, suggesting mechanisms other than *MDM2* genomic amplification as causal for *MDM2* overexpression (data not shown). Consequently, we investigated whether this *MDM2* over-expression is associated with the well-characterized promoter SNP 309 of *MDM2*, which influences expression from the P2 promoter of *MDM2* (Supplementary Figure 1a), we sequenced this SNP in 201 tumors and looked for correlations with *MDM2* expression and ploidy status where available (Supplementary Table 1, Supplementary Figure 1b–1e). SNP 309 TT, TG and GG genotypes were differentially associated with aneuploidy, although this did not reach statistical significance (*P* = 0.0926; Chi-square test). SNP 309 status also did not consistently correlate with overall levels of *MDM2* expression either by array-based analysis or in a small validation cohort of tumor RNA samples using qPCR (Supplementary Figure 1c–1d). No differences of *MDM2* P1 and P2 promoter usage, which is affected by SNP 309 (reviewed in [20]), were observed in exon-level RNA-sequencing analysis using a previously reported dataset [9] (Supplementary Figure 1e), suggesting other mechanisms upregulate *MDM2* in aneuploid PA tumors (Supplementary Figure 1a).

### *MDM2*-correlated gene signatures are robust in pilocytic astrocytoma

Utilizing the R2 database tool, we analyzed genome-wide gene expression correlations with *MDM2* (absolute correlation, FDR < 0.001) in PA and other gliomas to determine candidate programs that may lead to aneuploidy in these tumors. Several hundred genes correlated very strongly with the expression of *MDM2* within a PA dataset of 122 tumors profiled for ploidy status (741 genes at FDR < 0.001). Interestingly, in other publicly available glioma datasets including pediatric high-grade gliomas [21], DIPGs [22] and ependymomas [23], such correlations with *MDM2* expression do not exist, with very few genes passing the same false discovery rate criteria (FDR < 0.001) although this might be partially explained by PAs being very homogeneous entities whereas other tumor types are mixtures of multiple subgroups (Supplementary Figure 2a–2c). Gene Ontology (GO) analysis (Supplementary Table 2) revealed significant GO terms corresponding to protein ubiquitination during ubiquitin-dependent protein catabolic process (GO: 42787), DNA damage response, signal transduction by p53 class mediator resulting in cell cycle arrest (GO: 6977), CNS development (GO: 7417), ligase activity (GO: 16874), cell cycle arrest (GO: 7050), cell cycle checkpoint (GO: 75) and G1 phase of mitotic cell cycle (GO: 80) amongst others (Supplementary Table 2). Utilizing the R2 chromosome mapping tool we mapped the 741-gene signature to respective chromosomal positions to determine any specific enrichment across the genome. In contrast to the aneuploidy signature, correlated genes appeared to be over-represented across chromosome 19 (*P* = 4.0×10^−5^) and chromosome 12 (*P* = 7.5×10^−3^), but not any other chromosome.

### BRAF-fused PA tumors comprise molecular entities with distinct characteristics

We analyzed various clinical and molecular characteristics of tumors with *BRAF* fusions compared to tumors without *BRAF* duplications/fusions (Supplementary Table 3). Tumors harbouring *BRAF* fusions/duplications were much more likely to be located in the infratentorial compartment (*P* < 0.0001), to be wild-type for *FGFR1*/*BRAF* mutations (*P* < 0.0001), to be euploid (*P* = 0.0076) and to occur in children of younger mean age (11.1 years compared to 19.8 years). No statistically significant difference in progression-free survival was observed between patients with tumors positive or negative for *BRAF* duplication/fusion (Supplementary Figure 3, Supplementary Table 3), in keeping with data observed previously in adult PA patients [24].

**Figure 5:**
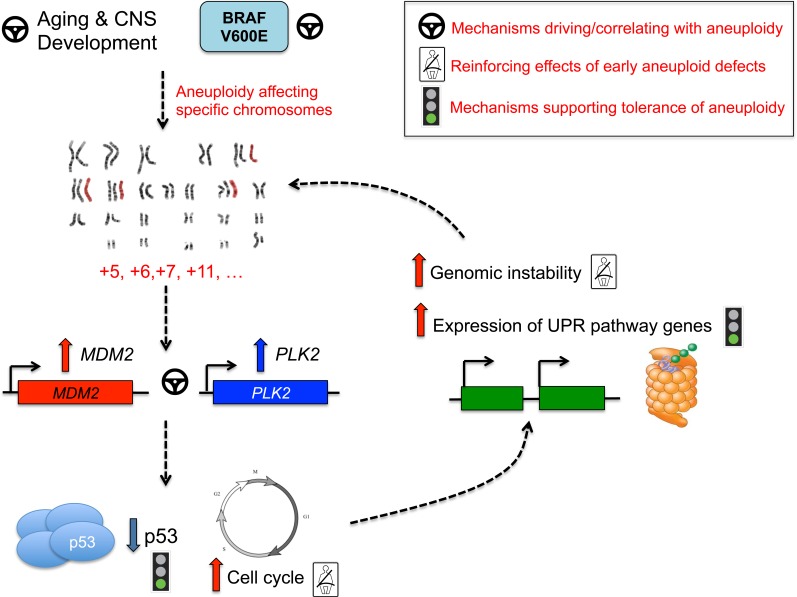
Pathway convergence leading to aneuploidy in PA

## DISCUSSION

We identify herein in a large cohort of 222 adult and pediatric PA located throughout the CNS, non-random chromosomal gains with a substantial enrichment of chromosomes 5, 6, 7 and 11. This aneuploidy genotype predominates in older patients with PA, in keeping with an earlier aCGH report on 44 PA samples [15]; mainly occurs in extra-cerebellar locations, and favors tumors that carry *BRAF* V600E but not *BRAF* fusions or *FGFR1* mutations. Aneuploid tumors had increased levels of *MDM2* and *PLK2* which are involved in cell-cycle arrest and ploidy control and may contribute to the observed genotype. Importantly, aneuploidy was identified in a large number of older patients with PA, and may represent an additional molecular driver in these samples.

Whole-chromosome alterations have been recurrently observed in several cancer types including mainly leukemias and sarcomas [25, 26]. Aneuploidy in brain tumors is considerably less well understood and has been under-explored. In normal brain, aneuploidy has been reported as a phenomenon of the aging mammalian CNS (reviewed in [27]). In the developing mouse brain, it affects various chromosomes in 33% of embryonic cortical [28] or postnatal subventricular zone [29] neuroblasts. Notably, aneuploidy is a common feature of the developed CNS [30] and displays a predilection to older mice [31], glial cells [31], specific chromosomes (murine chromosomes 7, 18 and Y), and the brain cortex [31], which shows significant increased age-related aneuploidy compared to the cerebellum (which remains euploid). These observations are consistent with our findings that aneuploid tumors are enriched in older patients and non-cerebellar areas, where *BRAF* fusions have the lowest incidence. However, we cannot formally exclude that some of the genomic alterations we see in adult PAs are not due to tumor environment differences associated with age, brain maturation and endocrine context. [8, 14].

BRAF expression and signaling is required for normal development of the cerebellum and hindbrain structures, and consequently cellular and murine studies of the *KIAA1549-BRAF* fusion show preferential growth selectivity in cerebellar neural stem cells [32, 33]. Within our dataset, aneuploid PA had significantly decreased frequency of *BRAF* fusion and mainly occurred outside cerebellar and hindbrain structures. They were, however, associated with higher frequencies of *BRAF* V600E, a genetic alteration enriched in PA located in extra-cerebellar regions. Interestingly, this mutation has been shown to induce aneuploidy through spindle anomalies and supernumerary centrosomes in melanocytic and melanoma cells [34–36]. Whether these opposing associations of *BRAF* fusion or activating mutation with aneuploidy in PAs is driven by subcellular expression patterns, brain-location specific differences or MAPK-independent functions based on brain regions will necessitate future mechanistic studies.

Aberrant regulation of developmental pathways may point to unique cellular origins of aneuploid PA tumor cells, or to developmental programs being re-activated from inherent early mammalian developmental stages which demonstrate aneuploidy and are proposed to underlie cellular diversity of the CNS (reviewed in[27]). Significance of pathways involved in the unfolded protein response and protein ubiquitination potentially illustrates that aneuploid PA tumors harbor mechanisms to tolerate stress accompanying extra chromosomal content. Indeed, chromosomal gains are accompanied by substantial cellular stress of increased gene and protein expression[37] and studies in yeast demonstrate that aneuploidy causes proteotoxic stress, including accumulation of proteins more challenging to fold[38]. Aneuploidy may thus represent a large-scale “stress-inducible mutation”[39]. The triggering of the unfolded protein binding pathways may thus represent a mechanism that aneuploid tumors use to adapt and permit unadulterated growth of aneuploid cells (Figure 5). Alterations in the cell cycle can contribute to mitotic checkpoint dysregulation, continued cell growth and ultimately aneuploid defects[40]. Polo-like kinases, including PLK1 and PLK2, are critical regulators of cell division[41–43] and PLK2 is required for proper centriole duplication [42–47]. In our dataset, PLK2 was over-expressed in aneuploid PA tumors, and presents an interesting candidate for promotion and reinforcement of aneuploidy. Many genes differentially regulated between aneuploid and euploid tumors in our dataset mapped to commonly trisomic chromosomes, with striking enrichment seen across chromosome 5 (*P*=2.50×10^−91^). With such high frequency, chromosome 5 gains are not only well-tolerated in PA tumors, but may actively reinforce aneuploid phenotypes based on candidate mapping of genes including *PLK2* (Figure 5). *MDM2* overexpression was also significantly associated with aneuploid PA and older patients. It strongly correlated with *PLK2* expression. *Mdm2* transgenic mice have increased age-related aneuploid defects affecting the rate of chromosome gain, but not loss[48, 49] similar to what is seen in our PA dataset. *MDM2* targets p53 for degradation and its amplification/overexpression mimics *TP53* loss[50, 51]. The p53 regulatory pathways plays a critical role in limiting the progression of aneuploidy and preserving the nature of diploid human cells [52]. There are no reported *TP53* alterations in PA [9, 12], but increased *MDM2* expression in aneuploid PA tumors may underlie inhibition of p53 function and subsequent continued growth of aneuploid cells. Our data show that a statistically robust 741-gene signature, correlated with *MDM2* expression, exists in a large dataset of 122 PA tumors, but not other pediatric gliomas. GO analysis of these *MDM2* correlated genes also points to cyclin-dependent kinases, cell cycle arrest and checkpoint pathways, ligase activity and CNS development (Supplementary Table 2). This data suggests a central role for *MDM2* mediated programs that may cause and/or tolerate aneuploidy in PA (reviewed in[40]) (Figure 5). Moreover, maintenance of aberrant p53 signaling through *PLK2* has been identified in previous studies[53], further suggesting an intriguing link between *PLK2* and *MDM2* overexpression and defective p53 cell cycle control that may further promote aneuploidy and tumor formation. Future functional studies in the context of mediating aneuploidy in PA are needed to validate these concepts.

In summary, with the involvement of potential driving, reinforcing and tolerating mechanisms of aneuploidy, we describe intriguing targets for both future mechanistic and diagnostic/therapeutic study of this phenotype and its role in (adult) PA tumorigenesis. As these adult PA show a reduced incidence of the most common genetic alterations associated with pediatric PA, this non-random aneuploidy may be an additional genetic modifier in this setting, and is potentially triggered by aging mechanisms in glial cells of the brain and further promoted by the molecular alterations we identify herein.

## MATERIALS AND METHODS

### Sample characteristics and pathological review

All samples were obtained with informed consent after approval of the Institutional Review Board of the respective hospitals they were treated in, and independently reviewed by senior pediatric neuropathologists (S.A., A.K., V.P.C., W.P. M.H.) according to the WHO guidelines. Despite central review, we cannot exclude that a small number of samples are not PA but represent other low grade gliomas. Patients and tumor samples are described in Supplementary Table 1. A total of 280 patient tumors were included in our cohort, with copy number data available for 222 tumors, and *KIAA1549-BRAF* fusion*/BRAF* duplication data available for the majority of cases (*n* = 268). *BRAF* mutation status was available for 231 tumors and *FGFR1* hot spot mutation data available for 122 samples as described previously. A large cohort of PA tumors (*n* = 126) was utilized for gene expression profiling, assembling data from newly processed samples and previously published profiles of well-characterized tumor samples [9], using the Affymetrix HG-U133 plus 2.0 microarray (Affymetrix). Out of this gene expression cohort, all tumors (*n* = 126) had age group information, *n* = 124 had *BRAF* fusion status, *n* = 108 had *BRAF* mutation status and *n* = 122 had available copy number information, with the techniques performed on individual samples described in Supplementary Table 1. Gene expression data was compared with other published datasets of glioblastoma (*n* = 84), pediatric high-grade glioma (*n* = 53), diffuse-intrinsic pontine glioma (DIPG) (*n* = 37), ependymoma (*n* = 83) normal brain [54] and normal cerebellum [55] as controls. Age≥18 is classified as adult. Both adult and pediatric tumors were obtained from the London/Ontario Tumor Bank, and from collaborators in Canada, Hungary, the United Kingdom and Germany.

### RNA and DNA extraction

RNA and DNA extraction methods for previously published cohorts are described in [6, 7, 9, 10, 15–17]. RNA and DNA were isolated from a subset of additional tumors using the Qiagen RNeasy Lipid Tissue Mini kit, Qiagen QIAmp/DNeasy DNA Mini kits respectively according to instructions of the manufacturer.

### KIAA1549-BRAF fusion screening

*BRAF* fusion status for previously described cohorts was determined as demonstrated in [6, 7, 9, 10, 16, 17]. RT-PCR to detect the three most common *KIAA1549-BRAF* fusion transcripts was performed for a subset of additional samples as previously described [6].

### DNA copy number analysis

For SNP analysis, DNA (250 ng) from PA samples was assayed with the HumanOmni1-Quad & Human Omni2.5 genotyping beadchip platforms according to the recommendations of the manufacturer (Illumina, San Diego, CA, USA). For a subset of formalin-fixed paraffin-embedded (FFPE)-derived DNA samples (*n* = 13, Supplementary Table 1), DNA was first rescued with the Illumina FFPE restore assay kit (Illumina, Inc) and subsequently hybridized to the Illumina Omni2.5-8 v1.0 chip. These BeadChip platforms enable whole-genome genotyping of respectively over 1,140,419 (Omni1-Quad) and 2,379,855 (Omni2.5) tagSNP markers derived from the 1,000 Genomes project, all three HapMap phases, and recently published studies. Image intensities were extracted using Illumina's BeadScan software. Data for each BeadChip were self-normalized using information contained within the array. Copy number data was also included from previously published datasets utilizing SNP arrays and array-based comparative genomic hybridization (aCGH) [6, 7, 10, 15, 16] with new analyses incorporating whole chromosome copy number profiling from 450K methylation arrays (Illumina) utilizing the R package ‘conumee’ (http://www.bioconductor.org/packages/release/bioc/html/conumee.html) essentially as described [56] and applied to published data [17]. In total, whole chromosomal copy number data was combined to yield a dataset of 222 tumors with numerical chromosome information to assess ploidy status.

### Gene expression profiling and analysis

A large dataset of PA tumors (*n* = 126) [9] was combined for gene expression profiling using the online R2: microarray analysis and visualization platform (http://r2.amc.nl). Ploidy status information was available for the majority of tumors with Affymetrix HG-U133 plus 2.0 gene expression data (*n* = 122) and was compared on this basis and on the basis of other molecular and clinical variables including *BRAF* fusion (data available for *n* = 124 tumors), *BRAF* mutation (*n* = 108 tumors) and age group (*n* = 126 tumors) through differential expression analysis using ANOVA at a false discovery rate (FDR) cut-off of < 0.001 (as are displayed in Figure 2c, Figure 3 and Figure 4). A list of 558 genes passing FDR criteria were kept and utilized for further analyses of the aneuploid phenotype. Gene Ontology (GO) analysis was performed for these 558 differentially expressed genes utilizing the R2 database tool and presented in Figure 3a. Gene expression of *MDM2* and *PLK2* was assessed using absolute correlation analysis with results displayed in Figure 3e. Based on its elevated expression in aneuploid PA tumors compared with other brain tumors and normal tissue, *MDM2* was pursued for additional correlation analyses. We investigated genes correlating (absolute correlation) with the expression of *MDM2* at a stringent false discovery rate (FDR) corrected criterion of < 0.001, which resulted in 741 robust combinations used for further Gene Ontology (GO) analysis and chromosome mapping in the dataset of 122 PA tumors profiled for aneuploidy. In control glioma and normal brain datasets detailed above, absolute correlation with identical FDR < 0.001 was calculated for genes correlating with *MDM2*. Representative heatmaps using transformed z-scores illustrating correlated signatures are displayed in Supplementary Figure 2a–2c and were generated utilizing the R2 database tool. Exonic level expression of exons 1a, 1b and 2 of *MDM2* from previously published RNA-sequencing data [9] was assessed to determine ratios of promoter 1 (P1) and P2 usage in a subset of PA tumors with available data (*n* = 69).

### MDM2 SNP 309 sanger sequencing

Sanger sequencing of the *MDM2* promoter SNP 309 was performed on 201 tumors using HotStart FastTaq KIT (Kapa) using forward primer: 5′-TGGTGAGGAGCAGGTACTGG-3′ and reverse primer: 5′-CGGAACGTGTCTGAACTTGA-3′ with the following PCR cycling conditions: 1 cycle of 1 minute at 96°C, 33 cycles of 10 seconds at 96°C, 5 seconds at 58°C and 1 second at 72°C followed by 1 cycle at 72°C for 30 seconds.

### Quantitative RT-PCR determination of MDM2 expression levels

Quantitative determination of *MDM2* expression levels was performed utilizing the Ssofast Evagreen kit (BioRad) with standard conditions indicated by the manufacturer at an annealing temperature of 58°C on the Roche LightCycler 480 (Roche) in a subset of 22 PA samples. Cycle threshold (C_t_) values were normalized to β-actin (*ACTB*) and a calibrator normal brain sample with wild-type (TT) *MDM2* SNP 309 genotype using the 2^−ΔΔCt^ method. The following primers were used:

5′-TCTCAAGCTCCGTGTTTGGTCAGT-3′, *MDM2* forward

5′-ACCTTGCAACAGCTGCAGATGAAC-3′, *MDM2* reverse

5′-GGCACCCAGCACAATGAAGATCAA-3′, β-actin forward 5′TAGAAGCATTTGCGGTGGACGATGGA-3′. β-actin reverse

### Statistical analyses

Contingency (Fisher's Exact Test), unpaired, two-tailed t-test comparisons and one-way ANOVA were performed utilizing GraphPad Prism to determine *P-*values. ANOVA, absolute correlation and multiple test correction using false discovery rate (FDR) and Gene Ontology (GO) calculations were performed within R2. Kaplan-Meier analyses were performed within GraphPad Prism for tumors with available data: aneuploidy (*n* = 193), *BRAF* fusion (*n* = 233), *BRAF* mutation (*n* = 196), brain location (*n* = 235).

## SUPPLEMENTARY MATERIAL FIGURES AND TABLES








