# Supplementary material for: Non-random aneuploidy specifies subgroups of pilocytic astrocytoma and correlates with older age
Source: Oncotarget. 2015 Sep 10;6(31):31844–56. doi: 10.18632/oncotarget.5571 (PMC4741644; doi:10.18632/oncotarget.5571)
Supplement: Supplementary file 1 [file oncotarget-06-31844-s001.pdf]

Non-random aneuploidy specifies subgroups of pilocytic astrocytoma and correlates with older age

Supplementary Material

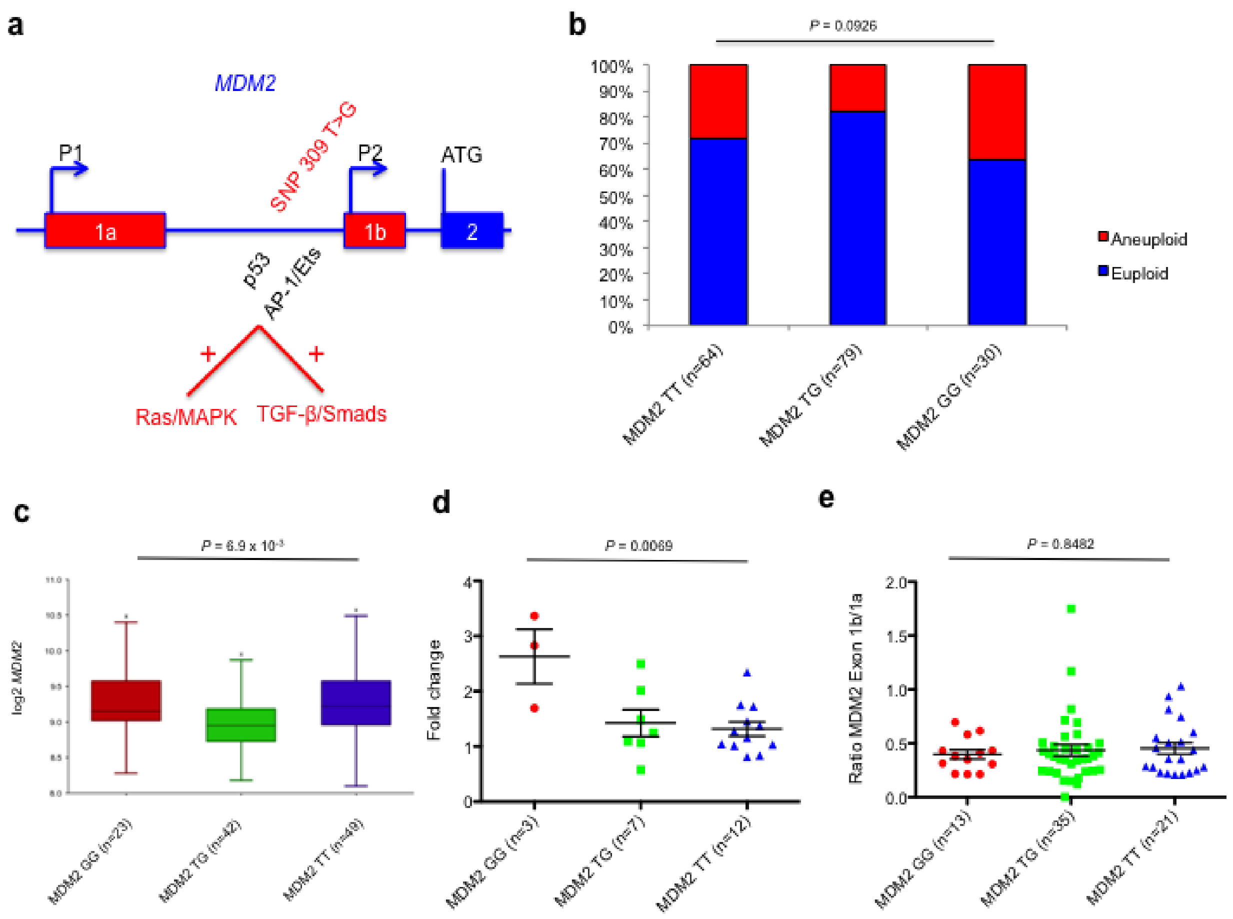

Supplementary Figure 1: Characterization of the *MDM2* promoter SNP 309 in PA tumors. **a,** Schematic depicting the *MDM2* promoter and mechanisms influencing promoter 2 (P2) usage and regulated *MDM2* expression. **b,** Frequency of aneuploidy amongst *MDM2* SNP 309 genotypes shows a trend toward differential association with this phenotype (P = 0.0926; Chi-square test). **c,** Gene expression analysis within array data with available MDM2 SNP 309 status (n=114) shows differential expression in association with SNP 309 genotypes ($P = 6.9 \times 10^{-3}$; ANOVA). **d,** qRT-PCR validation of *MDM2* expression in 22 samples compared by *MDM2* SNP

309 genotype shows approximately 3-fold elevated expression in *MDM2* GG-tumors (*P* = 0.0069; ANOVA). Fold changes values were calculated using the $2^{-\Delta\Delta Ct}$ method. **e,** RNA-seq derived ratios (from previously published RNA-seq profiles in [9]) of exonic level expression of exons 1a and 1b amongst *MDM2* SNP 309 genotyped PA tumors (n=69) demonstrates no association with genotype (*P* = 0.8482; ANOVA).

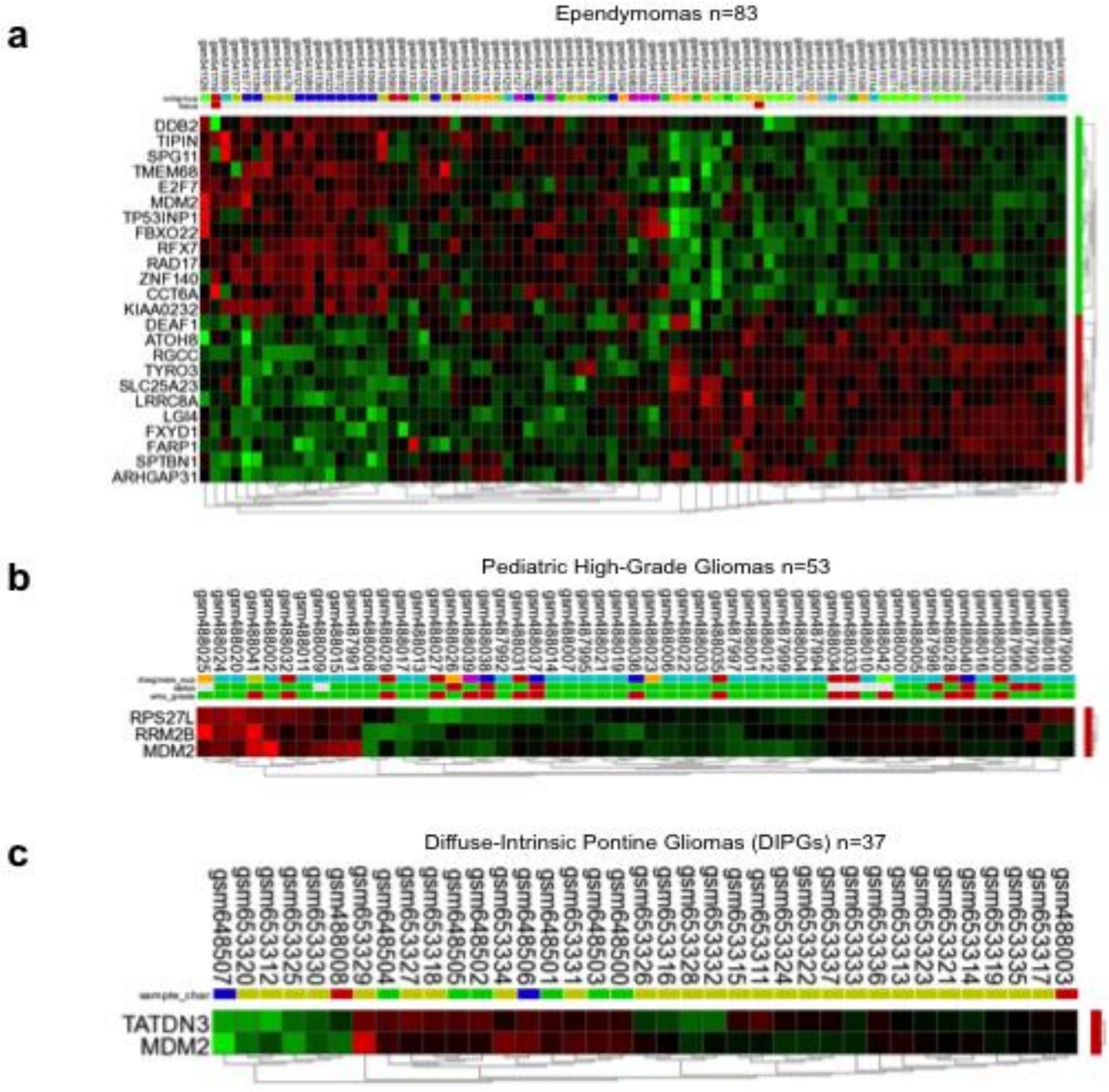

Supplementary Figure 2: *MDM2*-correlated pathways are not observed in non-PA gliomas.

Heatmaps of transformed z-scores of genes significantly correlated with *MDM2* expression in ependymomas **(a),** pediatric high-grade gliomas **(b)** and DIPGs **(c)** at FDR<0.001.

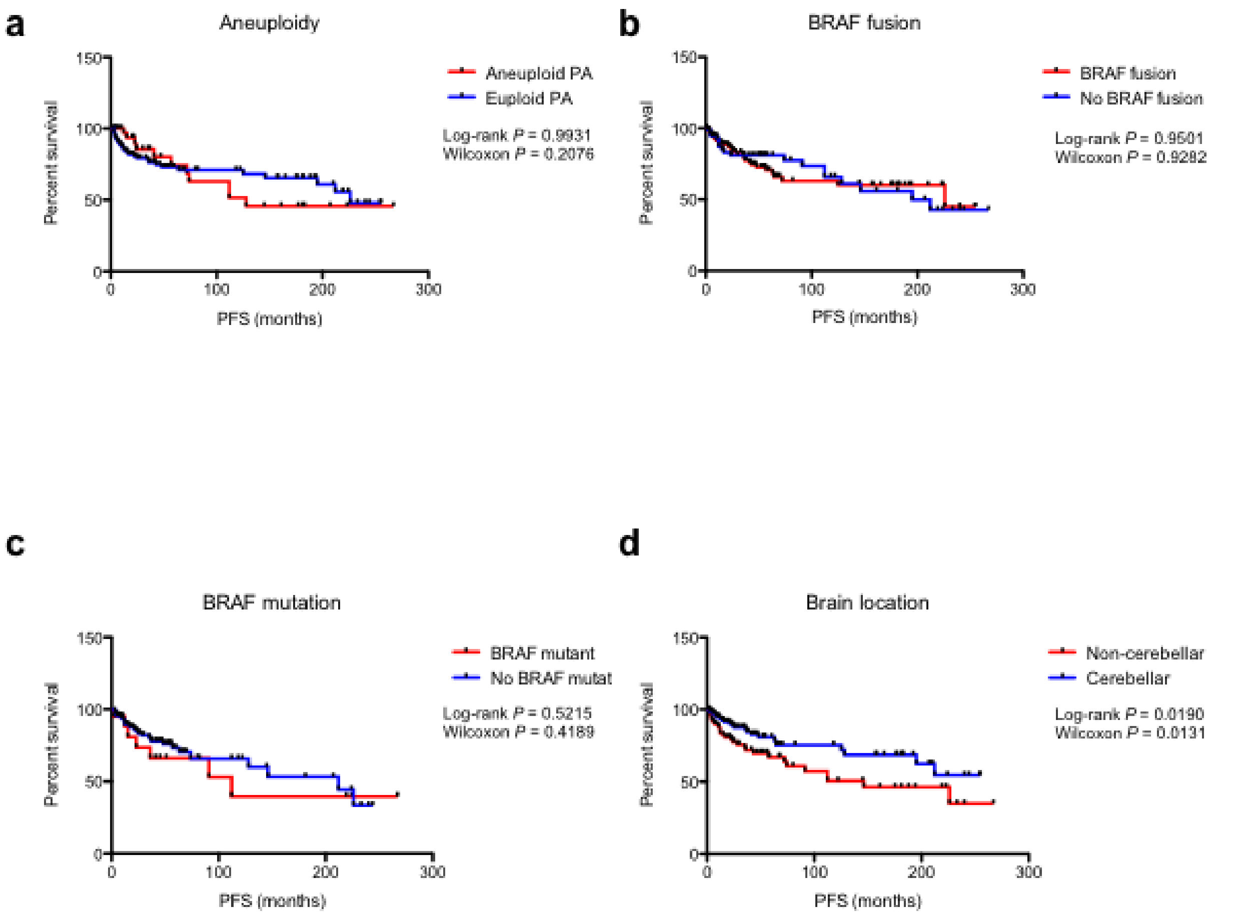

Supplementary Figure 3: Kaplan-Meier progression-free survival analysis of pilocytic astrocytoma patients included in the study. Kaplan-Meier progression-free survival (PFS, months) was assessed across different clinical and biological categories including aneuploidy (**a**), *BRAF* fusion (**b**), *BRAF* mutation (**c**) and brain location (**d**) with P-values calculated from Log-rank and Wilcoxon tests included in each panel.

Supplementary Table 1: Clinico-pathologic and molecular characteristics of pilocytic astrocytomas included in the study

Supplementary Table 2: Gene Ontology Analysis of 741 *MDM2*-correlated genes in 122 pilocytic astrocytoma samples with available aneuploid status at FDR<0.001

Supplementary Table 3: Characteristics of tumors with *BRAF* duplication/fusion included in the study
