# Supplementary material for: Non-random aneuploidy specifies subgroups of pilocytic astrocytoma and correlates with older age
Source: Oncotarget. 2015 Sep 10;6(31):31844–56. doi: 10.18632/oncotarget.5571 (PMC4741644; doi:10.18632/oncotarget.5571)
Supplement: Supplementary file 2 [file oncotarget-06-31844-s002.pdf]

Supplementary Table 1: Clinico-pathologic and molecular characteristics of pilocytic astrocytomas included in the study

Sample ID	Gender	Age at Diagnosis	Age Group	Specific Neuroanatomical Location	Infra/Supratentorial	Aneuploidy	KIAA1549-BRAF Fusion	FGFR1 mutation	PTPN11 mutation	BRAF	MDM2 SNP 309	PLK2 IHC	Gene Expression Array	Reference	CNV Data Platform	CNV Reference	Progression Free Survival (months)	Relapse (0: no; 1: yes)	Complete resection (0: sub-total; 1: gross-total)
PA1	NA	21	Adult	Cerebellum	Infratentorial	NA	N	NA	NA	WT	TG	NA	Y	Jones et al, Nat Genet 2013	NA	NA	52	0	1
PA2	NA	21	Adult	Cerebellum	Infratentorial	NA	KIAA1549-BRAF	NA	NA	WT	TT	NA	NA	NA	NA	NA	38	0	1
PA3	NA	27	Adult	Diencephal	Supratentorial	NA	KIAA1549-BRAF	NA	NA	WT	NA	NA	NA	NA	NA	NA	28	1	0
PA4	NA	18	Adult	Cerebral	Supratentorial	NA	N	NA	NA	V600E	NA	NA	NA	NA	NA	NA	41	0	0
PA5	NA	32	Adult	Cerebellum	Infratentorial	NA	KIAA1549-BRAF	NA	NA	WT	GG	NA	NA	NA	NA	NA	6	0	1
PA6	NA	56	Adult	Cerebellum	Infratentorial	NA	KIAA1549-BRAF	NA	NA	WT	TG	NA	NA	NA	NA	NA	52	0	1
PA7	NA	18	Adult	Cerebellum	Infratentorial	NONE	KIAA1549-BRAF	NA	NA	WT	TT	NA	NA	NA	aCGH	Cin et al, Acta Neuropathol 2011	41	0	1
PA8	NA	19	Adult	Cerebellum	Infratentorial	NONE	KIAA1549-BRAF	NA	NA	WT	TT	NA	Y	Jones et al, Nat Genet 2013	aCGH	Cin et al, Acta Neuropathol 2011	8	0	1
PA9	NA	22	Adult	Cerebellum	Infratentorial	NONE	KIAA1549-BRAF	NA	NA	WT	TG	NA	Y	Jones et al, Nat Genet 2013	aCGH	Cin et al, Acta Neuropathol 2011	16	0	1
PA10	NA	33	Adult	Cerebral	Supratentorial	NA	N	NA	NA	WT	NA	NA	NA	NA	NA	NA	6	0	0
PA11	NA	42	Adult	Cerebellum	Infratentorial	NA	KIAA1549-BRAF	NA	NA	WT	GG	NA	NA	NA	NA	NA	36	1	0
PA12	NA	22	Adult	Cerebral	Supratentorial	NA	N	WT	WT	ins598T	TG	NA	NA	NA	NA	NA	8	0	1
PA13	NA	40	Adult	Cerebellum	Infratentorial	NA	N	NA	NA	WT	NA	NA	NA	NA	NA	NA	63	0	1
PA14	F	33	Adult	Optic nerve	Supratentorial	NONE	N	WT	WT	WT	TG	NA	NA	NA	aCGH	Jones et al, J Neuropathol Exp Neurol 2006	240	0	NA
PA15	M	32	Adult	Cerebellum	Infratentorial	NONE	KIAA1549-BRAF	NA	NA	WT	TG	NA	NA	NA	aCGH	Jones et al, J Neuropathol Exp Neurol 2006	224	0	NA
PA16	F	20	Adult	Cerebellum	Infratentorial	NONE	KIAA1549 EXON 16-BRAF EXON 9	NA	NA	NA	TT	NA	Y	Jones et al, Nat Genet 2013	aCGH	Jones et al, J Neuropathol Exp Neurol 2006	224	0	NA
PA17	F	33	Adult	Pineal region	Supratentorial	NONE	N	NA	NA	WT	NA	NA	NA	NA	aCGH	Jones et al, J Neuropathol Exp Neurol 2006	146	1	NA
PA18	M	25	Adult	Cerebellum	Infratentorial	Gain: +5, +6, +7, +8, +11	KIAA1549 EXON 16-BRAF EXON 11	WT	WT	NA	TT	NA	Y	Jones et al, Nat Genet 2013	aCGH	Jones et al, J Neuropathol Exp Neurol 2006	181	0	NA
PA19	F	26	Adult	Right cerebellar hemisphere	Infratentorial	Gain: +5, +6, +7, +20	KIAA1549 EXON 16-BRAF EXON 9	NA	NA	NA	NA	NA	Y	Jones et al, Nat Genet 2013	aCGH	Jones et al, J Neuropathol Exp Neurol 2006	177	0	NA
PA20	F	20	Adult	Cerebellum	Infratentorial	Gain: +5, +6, +7, +8, +11, +15, +20	KIAA1549 EXON 16-BRAF EXON 9	WT	WT	NA	TG	NA	Y	Jones et al, Nat Genet 2013	aCGH	Jones et al, J Neuropathol Exp Neurol 2006	12	0	NA
PA21	F	23	Adult	4th ventricle	Infratentorial	Gain: +5, +6, +7, +10, +15, +20	N	NA	NA	NA	NA	NA	NA	NA	aCGH	Jones et al, J Neuropathol Exp Neurol 2006	112	1	NA
PA22	M	19	Adult	4th ventricle	Infratentorial	Gain: +5, +6, +11, +15, +20	KIAA1549 EXON 15-BRAF EXON 9	NA	NA	NA	NA	NA	NA	NA	aCGH	Jones et al, J Neuropathol Exp Neurol 2006	72	1	NA
PA23	M	21	Adult	Cerebellum	Infratentorial	NONE	N	NA	NA	WT	NA	NA	NA	NA	aCGH	Jones et al, J Neuropathol Exp Neurol 2006	244	0	NA
PA24	F	26	Adult	Cerebellum	Infratentorial	Gain: +5, +7, +9, +10, +11, +12, +13, +14, +15	KIAA1549 EXON 15-BRAF EXON 9	NA	NA	NA	TT	NA	Y	Jones et al, Nat Genet 2013	aCGH	Jones et al, J Neuropathol Exp Neurol 2006	41	1	NA
PA25	M	22	Adult	Brainstem	Infratentorial	NONE	KIAA1549 EXON 16-BRAF EXON 9	NA	NA	WT	TG	NA	Y	This study	SNP: Illumina Omni1-Quad	This study	226	1	NA
PA26	F	23	Adult	Cervicomedullar	Infratentorial	Gain: +5, +7	N	NA	WT	WT	TT	NA	NA	NA	SNP: Illumina Omni1-Quad	This study	74	1	NA
PA27	M	25	Adult	Cerebellum	Infratentorial	Gain: +6, +10, +12, +15	N	NA	NA	WT	TT	NA	Y	This study	SNP: Illumina Omni1-Quad	This study	35	0	1
PA28	M	29	Adult	Cerebellum	Infratentorial	NONE	N	NA	NA	WT	TG	Y	NA	NA	SNP: Illumina Omni1-Quad	This study	NA	NA	NA
PA29	M	21	Adult	Cerebellum	Infratentorial	NONE	KIAA1549 EXON 16-BRAF EXON 9	NA	NA	WT	GG	NA	Y	This study	SNP: Illumina Omni1-Quad	This study	NA	NA	NA
PA30	F	33	Adult	Cerebellum	Infratentorial	NONE	N	NA	NA	WT	GG	Y	Y	This study	SNP: Illumina Omni1-Quad	This study	0	0	0
PA31	F	65	Adult	Cerebellum	Infratentorial	Gain: +5, +6, +11, +12, +15	KIAA1549 EXON 15-BRAF EXON 9	NA	NA	WT	GG	Y	Y	This study	SNP: Illumina Omni1-Quad	This study	0	0	NA
PA32	F	19	Adult	Thalamus	Supratentorial	Gain: +2, +4, +5, +6, +7, +8, +10, +11, +14, +15, +16, +17, +18, +19, +20, +21, +22, +X	N	WT	WT	V600E	TT	NA	Y	This study	SNP: Illumina Omni1-Quad	This study	112	1	NA
PA33	M	45	Adult	Cerebrum	Supratentorial	Gain: +7	KIAA1549-BRAF	WT	WT	WT	GG	NA	Y	This study	SNP: Illumina Omni1-Quad	This study	0	0	1
PA34	M	20	Adult	Cerebrum	Supratentorial	Gain: +5, +6, +7, +9, +10, +12,+20	N	WT	WT	WT	GG	Y	Y	This study	SNP: Illumina Omni1-Quad	This study	0	0	1
PA35	F	45	Adult	Cerebrum	Supratentorial	Gain: +5, +7, +8, +11	N	WT	WT	WT	TT	Y	Y	This study	SNP: Illumina Omni1-Quad	This study	0	0	1
PA36	F	34	Adult	Cerebellum	Infratentorial	NA	N	NA	NA	WT	NA	NA	NA	NA	NA	NA	0	0	0
PA37	F	32	Adult	Cerebellum	Infratentorial	NA	KIAA1549 EXON 16-BRAF EXON 9	NA	NA	WT	TG	NA	NA	NA	NA	NA	NA	NA	NA
PA38	M	23	Adult	Cerebrum	Supratentorial	NA	N	Intronic: chr8: 38272435 C>T	WT	WT	TG	NA	NA	NA	NA	NA	112	0	NA
PA39	NA	5	Pediatric	Cerebellum	Infratentorial	NONE	KIAA1549-BRAF	NA	NA	WT	TT	NA	Y	Jones et al, Nat Genet 2013	aCGH	Cin et al, Acta Neuropathol 2011	38	0	1
PA40	NA	4	Pediatric	Cerebellum	Infratentorial	NONE	KIAA1549-BRAF	NA	NA	WT	TG	NA	Y	Jones et al, Nat Genet 2013	aCGH	Cin et al, Acta Neuropathol 2011	48	0	1
PA41	NA	10	Pediatric	Cerebellum	Infratentorial	NONE	KIAA1549-BRAF	NA	NA	WT	TG	NA	Y	Jones et al, Nat Genet 2013	aCGH	Cin et al, Acta Neuropathol 2011	54	0	1
PA42	NA	15	Pediatric	Cerebellum	Infratentorial	NA	KIAA1549-BRAF	NA	NA	WT	NA	NA	NA	NA	NA	NA	37	1	0
PA43	NA	13	Pediatric	Cerebellum	Infratentorial	NA	SRGAP3-RAF1	NA	NA	WT	NA	NA	NA	NA	NA	NA	30	0	1
PA44	NA	9	Pediatric	Cerebellum	Infratentorial	NA	KIAA1549-BRAF	NA	NA	WT	NA	NA	NA	NA	NA	NA	63	0	0
PA45	NA	4	Pediatric	Cerebellum	Infratentorial	NONE	KIAA1549-BRAF	NA	NA	WT	TT	NA	Y	Jones et al, Nat Genet 2013	aCGH	Cin et al, Acta Neuropathol 2011	54	0	1
PA46	NA	6	Pediatric	Cerebellum	Infratentorial	NONE	KIAA1549-BRAF	NA	NA	WT	TG	NA	Y	Jones et al, Nat Genet 2013	aCGH	Cin et al, Acta Neuropathol 2011	28	0	1
PA47	NA	8	Pediatric	Cerebellum	Infratentorial	NA	SRGAP3-RAF1	NA	NA	WT	NA	NA	NA	NA	NA	NA	44	0	1
PA48	NA	9	Pediatric	Cerebellum	Infratentorial	NONE	KIAA1549-BRAF	NA	NA	WT	GG	NA	Y	Jones et al, Nat Genet 2013	aCGH	Cin et al, Acta Neuropathol 2011	38	0	1
PA49	NA	12	Pediatric	Cerebellum	Infratentorial	NA	KIAA1549-BRAF	NA	NA	WT	NA	NA	NA	NA	NA	NA	47	0	1
PA50	NA	17	Pediatric	Cerebral	Supratentorial	NA	N	NA	NA	WT	NA	NA	Y	Jones et al, Nat Genet 2013	NA	NA	6	0	1
PA51	NA	7	Pediatric	Cerebellum	Infratentorial	NA	KIAA1549-BRAF	NA	NA	V600E	NA	NA	NA	NA	NA	NA	45	0	1
PA52	NA	6	Pediatric	Cerebellum	Infratentorial	NA	N	NA	NA	WT	NA	NA	NA	NA	NA	NA	4	0	1
PA53	NA	4	Pediatric	Cerebellum	Infratentorial	Gain: +5, +6, +7, +8, +11, +12, +18, +20	KIAA1549-BRAF	NA	NA	WT	GG	NA	Y	Jones et al, Nat Genet 2013	aCGH	Cin et al, Acta Neuropathol 2011	20	0	0
PA54	NA	16	Pediatric	Cerebral	Supratentorial	NA	N	NA	NA	WT	NA	NA	NA	NA	NA	NA	50	0	1
PA55	NA	17	Pediatric	Cerebellum	Infratentorial	NA	N	NA	NA	WT	NA	NA	NA	NA	NA	NA	10	0	0
PA56	NA	8	Pediatric	Cerebellum	Infratentorial	NA	N	NA	NA	WT	NA	NA	NA	NA	NA	NA	16	0	1
PA57	NA	16	Pediatric	Cerebral	Supratentorial	NONE	FAM131B-BRAF	NA	NA	WT	GG	NA	Y	Jones et al, Nat Genet 2013	aCGH	Cin et al, Acta Neuropathol 2011	68	0	1
PA58	NA	2	Pediatric	Cerebellum	Infratentorial	NONE	KIAA1549-BRAF	NA	NA	WT	NA	NA	Y	Jones et al, Nat Genet 2013	aCGH	Cin et al, Acta Neuropathol 2011	65	0	0
PA59	NA	11	Pediatric	Cerebellum	Infratentorial	NONE	KIAA1549-BRAF	NA	NA	WT	TT	NA	Y	Jones et al, Nat Genet 2013	aCGH	Cin et al, Acta Neuropathol 2011	4	0	1
PA60	NA	14	Pediatric	Diencephal	Supratentorial	NA	N	NA	NA	WT	NA	NA	NA	NA	NA	NA	52	0	1
PA61	NA	13	Pediatric	Diencephal	Supratentorial	NA	KIAA1549-BRAF	NA	NA	WT	NA	NA	NA	NA	NA	NA	67	0	1
PA62	NA	17	Pediatric	Cerebellum	Infratentorial	NA	KIAA1549-BRAF	NA	NA	WT	NA	NA	NA	NA	NA	NA	36	0	1
PA63	NA	16	Pediatric	Cerebellum	Infratentorial	NONE	KIAA1549-BRAF	NA	NA	WT	TT	NA	Y	Jones et al, Nat Genet 2013	aCGH	Cin et al, Acta Neuropathol 2011	9	1	0
PA64	NA	14	Pediatric	Cerebral	Supratentorial	NONE	N	NA	NA	V600E	TT	NA	Y	Jones et al, Nat Genet 2013	aCGH	Cin et al, Acta Neuropathol 2011	80	0	0
PA65	NA	12	Pediatric	Cerebellum	Infratentorial	NA	N	NA	NA	WT	NA	NA	NA	NA	NA	NA	53	0	1
PA66	NA	13	Pediatric	Cerebral	Supratentorial	NA	N	NA	NA	WT	NA	NA	NA	NA	NA	NA	24	0	0
PA67	NA	13	Pediatric	Cerebellum	Infratentorial	NA	KIAA1549-BRAF	NA	NA	WT	NA	NA	NA	NA	NA	NA	48	0	1
PA68	NA	13	Pediatric	Cerebellum	Infratentorial	Gain: +5, +6, +11, +21	KIAA1549-BRAF	NA	NA	WT	TT	NA	Y	Jones et al, Nat Genet 2013	aCGH	Cin et al, Acta Neuropathol 2011	37	0	1
PA69	NA	6	Pediatric	Brain stem	Infratentorial	Gain: +6, +7, +15	KIAA1549-BRAF	NA	NA	WT	GG	NA	NA	NA	aCGH	Cin et al, Acta Neuropathol 2011	24	1	0
PA70	NA	11	Pediatric	Cerebellum	Infratentorial	Gain: +5, +7	KIAA1549-BRAF	NA	NA	WT	GG	NA	Y	Jones et al, Nat Genet 2013	aCGH	Cin et al, Acta Neuropathol 2011	8	0	1
PA71	NA	12	Pediatric	Cerebellum	Infratentorial	NA	KIAA1549-BRAF	NA	NA	WT	NA	NA	NA	NA	NA	NA	63	1	0
PA72	NA	3	Pediatric	Diencephal	Supratentorial	NONE	KIAA1549-BRAF	NA	NA	V600E	TT	NA	Y	Jones et al, Nat Genet 2013	aCGH	Cin et al, Acta Neuropathol 2011	36	f	0
PA73	NA	17	Pediatric	Cerebellum	Infratentorial	Gain: +5, +7, +8, +18	N	NA	NA	ins598T	TT	NA	Y	Jones et al, Nat Genet 2013	aCGH	Cin et al, Acta Neuropathol 2011	23	1	0
PA74	NA	12	Pediatric	Diencephal	Supratentorial	NONE	KIAA1549-BRAF	NA	NA	WT	TG	NA	Y	Jones et al, Nat Genet 2013	aCGH	Cin et al, Acta Neuropathol 2011	24	0	0
PA75	NA	4	Pediatric	Diencephal	Supratentorial	Gain: +11	N	WT	WT	V600E	TG	NA	NA	NA	aCGH	Cin et al, Acta Neuropathol 2011	12	1	0
PA76	NA	11	Pediatric	Cerebellum	Infratentorial	Gain: +5, +6, +7, +8	N	NA	NA	WT	TG	NA	Y	Jones et al, Nat Genet 2013	aCGH	Cin et al, Acta Neuropathol 2011	30	0	1
PA77	NA	5	Pediatric	Cerebellum	Infratentorial	NA	KIAA1549-BRAF	NA	NA	WT	NA	NA	NA	NA	NA	NA	14	0	1
PA78	NA	2	Pediatric	Cerebellum	Infratentorial	NA	KIAA1549-BRAF	NA	NA	WT	NA	NA	NA	NA	NA	NA	54	0	1
PA79	NA	10	Pediatric	Cerebellum	Infratentorial	NONE	KIAA1549-BRAF	NA	NA	WT	TT	NA	Y	Jones et al, Nat Genet 2013	aCGH	Cin et al, Acta Neuropathol 2011	25	1	0
PA80	NA	3	Pediatric	Diencephal	Supratentorial	NONE	N	WT	WT	V600E	TT	NA	Y	Jones et al, Nat Genet 2013	aCGH	Cin et al, Acta Neuropathol 2011	51	0	0
PA81	NA	2	Pediatric	Cerebellum	Infratentorial	NONE	KIAA1549-BRAF	NA	NA	WT	TG	NA	Y	Jones et al, Nat Genet 2013	aCGH	Cin et al, Acta Neuropathol 2011	48	1	1
PA82	NA	4	Pediatric	Cerebellum	Infratentorial	NONE	KIAA1549-BRAF	NA	NA	WT	TG	NA	Y	Jones et al, Nat Genet 2013	aCGH	Cin et al, Acta Neuropathol 2011	8	0	1
PA83	NA	6	Pediatric	Cerebellum	Infratentorial	NONE	KIAA1549-BRAF	NA	NA	WT	TT	NA	Y	Jones et al, Nat Genet 2013	aCGH	Cin et al, Acta Neuropathol 2011	60	0	1
PA84	NA	5	Pediatric	Cerebellum	Infratentorial	NONE	KIAA1549-BRAF	NA	NA	WT	TT	NA	Y	Jones et al, Nat Genet 2013	aCGH	Cin et al, Acta Neuropathol 2011	16	1	0
PA85	NA	14	Pediatric	Cerebral	Supratentorial	NONE	KIAA1549-BRAF	NA	NA	WT	TT	NA	NA	NA	aCGH	Cin et al, Acta Neuropathol 2011	55	0	1
PA86	NA	8	Pediatric	Brain stem	Infratentorial	NA	N	NA	NA	WT	NA	NA	NA	NA	NA	NA	30	0	0
PA87	NA	10	Pediatric	Brain stem	Infratentorial	NA	KIAA1549-BRAF	NA	NA	WT	NA	NA	NA	NA	NA	NA	56	0	0
PA88	NA	7	Pediatric	Brain stem	Infratentorial	Gain: +19	KIAA1549-BRAF	NA	NA	WT	TT	NA	Y	Jones et al, Nat Genet 2013	aCGH	Cin et al, Acta Neuropathol 2011	57	1	0
PA89	NA	8	Pediatric	Cerebral	Supratentorial	NONE	KIAA1549-BRAF	NA	NA	WT	TG	NA	Y	Jones et al, Nat Genet 2013	aCGH	Cin et al, Acta Neuropathol 2011	58	0	0
PA90	NA	7	Pediatric	Cerebellum	Infratentorial	NONE	KIAA1549-BRAF	NA	NA	WT	TG	NA	NA	NA	aCGH	Cin et al, Acta Neuropathol 2011	41	0	1
PA91	NA	6	Pediatric	Cerebellum	Infratentorial	NONE	KIAA1549-BRAF	NA	NA	WT	TG	NA	NA	NA	aCGH	Cin et al, Acta Neuropathol 2011	70	0	1
PA92	NA	10	Pediatric	Cerebellum	Infratentorial	NONE	N	NA	NA	WT	TG	NA	NA	NA	aCGH	Cin et al, Acta Neuropathol 2011	12	1	0
PA93	NA	4	Pediatric	Diencephal	Supratentorial	NA	N	NA	NA	WT	NA	NA	NA	NA	NA	NA	12	1	0
PA94	M	5	Pediatric	Cerebellum	Infratentorial	NONE	KIAA1549 EXON 16-BRAF EXON 9	NA	NA	NA	NA	NA	NA	NA	aCGH	Jones et al, J Neuropathol Exp Neurol 2006	52	0	0
PA95	F	3	Pediatric	Cerebellum	Infratentorial	NONE	KIAA1549 EXON 16-BRAF EXON 9	NA	NA	NA	NA	NA	Y	Jones et al, Nat Genet 2013	aCGH	Jones et al, J Neuropathol Exp Neurol 2006	64	1	0
PA96	F	3	Pediatric	Optic nerve	Supratentorial	NONE	N	WT	WT	V600E	TT	NA	Y	Jones et al, Nat Genet 2013	aCGH	Jones et al, J Neuropathol Exp Neurol 2006	219	0	NA
PA97	F	8	Pediatric	Cerebellum	Infratentorial	NONE	KIAA1549 EXON 16-BRAF EXON 9	WT	WT	NA	TG	NA	Y	Jones et al, Nat Genet 2013	aCGH	Jones et al, J Neuropathol Exp Neurol 2006	1	0	NA
PA98	M	12	Pediatric	Hypothalamus	Supratentorial	Gain: +7	KIAA1549 EXON 16-BRAF EXON 9	WT	WT	NA	GG	NA	Y	Jones et al, Nat Genet 2013	aCGH	Jones et al, J Neuropathol Exp Neurol 2006	223	0	NA
PA99	F	17	Pediatric	Cerebellum	Infratentorial	NONE	N	NA	NA	insTAC	NA	NA	NA	NA	aCGH	Jones et al, J Neuropathol Exp Neurol 2006	224	0	NA
PA100	M	5	Pediatric	Cerebellum	Infratentorial	NONE	KIAA1549 EXON 16-BRAF EXON 9	NA	NA	NA	NA	NA	NA	NA	aCGH	Jones et al, J Neuropathol Exp Neurol 2006	240	0	1
PA101	F	14	Pediatric	Cerebellum	Infratentorial	Gain: +7	N	WT	WT	WT	TT	NA	Y	Jones et al, Nat Genet 2013	aCGH	Jones et al, J Neuropathol Exp Neurol 2006	207	0	NA
PA102	M	1	Pediatric	Hypothalamus	Supratentorial	NONE	N	NA	NA	WT	NA	NA	NA	NA	aCGH	Jones et al, J Neuropathol Exp Neurol 2006	233	0	0
PA103	M	13	Pediatric	3rd ventricle	Supratentorial	NONE	N	NA	NA	NA	NA	NA	NA	NA	aCGH	Jones et al, J Neuropathol Exp Neurol 2006	1	0	1
PA104	M	6	Pediatric	Cerebellum	Infratentorial	NONE	N	NA	NA	NA	NA	NA	NA	NA	aCGH	Jones et al, J Neuropathol Exp Neurol 2006	195	1	NA
PA105	F	5	Pediatric	4th ventricle	Infratentorial	NONE	KIAA1549 EXON 16-BRAF EXON 9	NA	NA	NA	NA	NA	NA	NA	aCGH	Jones et al, J Neuropathol Exp Neurol 2006	43	1	NA
PA106	M	9	Pediatric	4th ventricle	Infratentorial	NONE	KIAA1549 EXON 16-BRAF EXON 9	NA	NA	NA	NA	NA	Y	Jones et al, Nat Genet 2013	aCGH	Jones et al, J Neuropathol Exp Neurol 2006	188	0	NA
PA107	M	10	Pediatric	Optic nerve	Supratentorial	Gain: +5, +7, +9, +10, +15	N	NA	NA	V600E	NA	NA	NA	NA	aCGH	Jones et al, J Neuropathol Exp Neurol 2006	267	0	NA
PA108	F	13	Pediatric	Cerebellum	Infratentorial	Gain: +7	N	NA	NA	WT	NA	NA	NA	NA	aCGH	Jones et al, J Neuropathol Exp Neurol 2006	128	1	NA
PA109	M	4	Pediatric	Right temporal lobe	Supratentorial	NONE	KIAA1549 EXON 15-BRAF EXON 9	NA	NA	NA	NA	NA	NA	NA	aCGH	Jones et al, J Neuropathol Exp Neurol 2006	15	0	0
PA110	M	4	Pediatric	Cerebellum	Infratentorial	NONE	KIAA1549 EXON 16-BRAF EXON 9	WT	WT	NA	TG	NA	Y	Jones et al, Nat Genet 2013	aCGH	Jones et al, J Neuropathol Exp Neurol 2006	176	0	NA
PA111	M	2	Pediatric	3rd ventricle	Supratentorial	NONE	KIAA1549 EXON 16-BRAF EXON 9	NA	NA	NA	TT	NA	NA	NA	aCGH	Jones et al, J Neuropathol Exp Neurol 2006	175	0	NA
PA112	M	16	Pediatric	Cerebellum	Infratentorial	NONE	KIAA1549 EXON 16-BRAF EXON 9	NA	NA	NA	NA	NA	Y	Jones et al, Nat Genet 2013	aCGH	Jones et al, J Neuropathol Exp Neurol 2006	165	0	NA
PA113	F	11	Pediatric	Cerebellum	Infratentorial	Gain: +5	KIAA1549 EXON 16-BRAF EXON 9	WT	WT	NA	GG	NA	Y	Jones et al, Nat Genet 2013	aCGH	Jones et al, J Neuropathol Exp Neurol 2006	183	0	NA
PA114	M	4	Pediatric	Optic nerve	Supratentorial	NONE	KIAA1549 EXON 15-BRAF EXON 9	NA	NA	NA	NA	NA	NA	NA	aCGH	Jones et al, J Neuropathol Exp Neurol 2006	194	0	0
PA115	F	12	Pediatric	Left frontal lobe	Supratentorial	Gain: +4, +5, +7, +12	N	NA	NA	NA	NA	NA	Y	Jones et al, Nat Genet 2013	aCGH	Jones et al, J Neuropathol Exp Neurol 2006	161	0	NA
PA116	M	0.5	Pediatric	Optic nerve	Supratentorial	NONE	KIAA1549 EXON 15-BRAF EXON 9	NA	NA	NA	NA	NA	NA	NA	aCGH	Jones et al, J Neuropathol Exp Neurol 2006	3	1	NA
PA117	M	1	Pediatric	Optic nerve	Supratentorial	NONE	KIAA1549 EXON 15-BRAF EXON 9	NA	NA	NA	NA	NA	NA	NA	aCGH	Jones et al, J Neuropathol Exp Neurol 2006	7	1	NA
PA118	M	9	Pediatric	Cerebellum	Infratentorial	NONE	KIAA1549 EXON 16-BRAF EXON 9	WT	WT	NA	TG	NA	NA	NA	aCGH	Jones et al, J Neuropathol Exp Neurol 2006	158	0	NA
PA119	F	8	Pediatric	Cerebellum	Infratentorial	NONE	KIAA1549 EXON 16-BRAF EXON 9	NA	NA	NA	NA	NA	NA	NA	aCGH	Jones et al, J Neuropathol Exp Neurol 2006	157	0	NA
PA120	F	5	Pediatric	Cerebellum	Infratentorial	NONE	KIAA1549 EXON 16-BRAF EXON 9	NA	NA	NA	NA	NA	NA	NA	aCGH	Jones et al, J Neuropathol Exp Neurol 2006	182	0	NA
PA121	M	3	Pediatric	Cerebellum	Infratentorial	NONE	KIAA1549 EXON 16-BRAF EXON 11	NA	NA	NA	NA	NA	NA	NA	aCGH	Jones et al, J Neuropathol Exp Neurol 2006	255	0	NA
PA122	M	8	Pediatric	Right cerebellar hemisphere	Infratentorial	NONE	KIAA1549 EXON 16-BRAF EXON 9	NA	NA	NA	NA	NA	NA	NA	aCGH	Jones et al, J Neuropathol Exp Neurol 2006	192	0	NA
PA123	F	6	Pediatric	Cerebellum	Infratentorial	NONE	KIAA1549 EXON 15-BRAF EXON 9	NA	NA	NA	NA	NA	NA	NA	aCGH	Jones et al, J Neuropathol Exp Neurol 2006	125	1	NA
PA124	F	9	Pediatric	Cerebellum	Infratentorial	NONE	KIAA1549 EXON 16-BRAF EXON 9	WT	WT	NA	TG	NA	Y	Jones et al, Nat Genet 2013	aCGH	Jones et al, J Neuropathol Exp Neurol 2006	254	0	NA
PA125	F	5	Pediatric	Brain stem	Infratentorial	NONE	N	NA	NA	WT	NA	NA	NA	NA	aCGH	Jones et al, J Neuropathol Exp Neurol 2006	181	0	0
PA126	M	3	Pediatric	Cerebellum	Infratentorial	NONE	KIAA1549 EXON 16-BRAF EXON 9	NA	NA	NA	NA	NA	Y	Jones et al, Nat Genet 2013	aCGH	Jones et al, J Neuropathol Exp Neurol 2006	210	0	NA
PA127	F	2	Pediatric	Cerebellum	Infratentorial	NONE	KIAA1549 EXON 16-BRAF EXON 9	NA	NA	WT	TT	NA	NA	NA	SNP: Illumina Omni1-Quad	This study	68	0	1
PA128	F	12	Pediatric	Cerebellum	Infratentorial	Gain: +5, +6, +7, +11, +15, +16, +X	N	NA	NA	WT	TT	Y	Y	This study	SNP: Illumina Omni1-Quad	This study	10	0	0
PA129	M	1	Pediatric	Cerebellum	Infratentorial	NONE	N	NA	NA	WT	TG	Y	Y	This study	SNP: Illumina Omni1-Quad	This study	212	1	0
PA130	M	8	Pediatric	Optic Pathway	Supratentorial	Gain: +7	N	WT	WT	V600E	GG	Y	Y	This study	SNP: Illumina Omni1-Quad	This study	15	1	0
PA131	NA	4	Pediatric	Cerebellum	Infratentorial	NONE	KIAA1549-BRAF	NA	NA	NA	NA	NA	NA	NA	SNP: Illumina Omni1-Quad	Jacob et al, Br J Cancer 2009	NA	NA	NA
PA132	NA	4	Pediatric	Cerebellum	Infratentorial	NONE	KIAA1549-BRAF	NA	NA	WT	TT	NA	NA	NA	SNP: Illumina Omni1-Quad	Jacob et al, Br J Cancer 2009	NA	NA	NA
PA133	NA	4	Pediatric	Cerebellum	Infratentorial	NONE	N	NA	NA	WT	NA	NA	NA	NA	SNP: Illumina Omni1-Quad	Jacob et al, Br J Cancer 2009	NA	NA	NA
PA134	NA	4	Pediatric	Cerebellum	Infratentorial	NONE	KIAA1549-BRAF	NA	NA	WT	TT	NA	NA	NA	SNP: Illumina Omni1-Quad	Jacob et al, Br J Cancer 2009	NA	NA	NA
PA135	NA	10	Pediatric	Parietal lobe	Supratentorial	NONE	KIAA1549-BRAF	NA	NA	WT	NA	NA	NA	NA	SNP: Illumina Omni1-Quad	Jacob et al, Br J Cancer 2009	NA	NA	NA
PA136	NA	3	Pediatric	Hypothalamus	Supratentorial	NONE	KIAA1549-BRAF	NA	NA	WT	NA	NA	NA	NA	SNP: Illumina Omni1-Quad	Jacob et al, Br J Cancer 2009	NA	NA	NA
PA137	NA	4	Pediatric	Cerebellum	Infratentorial	NONE	KIAA1549-BRAF	NA	NA	WT	NA	NA	NA	NA	SNP: Illumina Omni1-Quad	Jacob et al, Br J Cancer 2009	NA	NA	NA

**PA138**	NA	4	Pediatric	Cerebellum	Infratentorial	Gain: +7, +17	KIAA1549-BRAF	NA	NA	WT	TG	NA	NA	NA	SNP: Illumina Omni1-Quad	Jacob et al, Br J Cancer 2009	NA	NA	NA
**PA139**	NA	1	Pediatric	Cervicomedullar	Infratentorial	NONE	NA	WT	WT	NA	TT	NA	Y	This study	SNP: Illumina Omni1-Quad	This study	3	1	0
**PA140**	NA	3	Pediatric	Cerebellum	Infratentorial	NONE	NA	NA	NA	NA	GG	NA	NA	NA	SNP: Illumina Omni1-Quad	Jacob et al, Br J Cancer 2009	NA	NA	NA
**PA141**	NA	6	Pediatric	Cerebellum	Infratentorial	NONE	NA	NA	NA	NA	NA	NA	NA	NA	SNP: Illumina Omni1-Quad	Jacob et al, Br J Cancer 2009	NA	NA	NA
**PA142**	NA	11	Pediatric	Cerebellum	Infratentorial	NONE	NA	NA	NA	NA	TT	NA	NA	NA	SNP: Illumina Omni1-Quad	Jacob et al, Br J Cancer 2009	NA	NA	NA
**PA143**	NA	13	Pediatric	Occipital lobe	Supratentorial	NONE	N	WT	WT	NA	TG	NA	NA	NA	SNP: Illumina Omni1-Quad	Jacob et al, Br J Cancer 2009	NA	NA	NA
**PA144**	NA	11	Pediatric	Cerebellum	Infratentorial	NONE	NA	NA	NA	NA	TT	NA	NA	NA	SNP: Illumina Omni1-Quad	Jacob et al, Br J Cancer 2009	NA	NA	NA
**PA145**	NA	9	Pediatric	Brainstem	Infratentorial	NONE	NA	WT	WT	NA	TG	NA	NA	NA	SNP: Illumina Omni1-Quad	Jacob et al, Br J Cancer 2009	NA	NA	NA
**PA146**	NA	4	Pediatric	Brainstem	Infratentorial	NONE	NA	WT	WT	NA	TT	NA	Y	This study	SNP: Illumina Omni1-Quad	This study	NA	NA	NA
**PA147**	M	16	Pediatric	Thalamus	Supratentorial	NA	N	WT	WT	V600E	TG	NA	Y	This study	NA	NA	0	1	NA
**PA148**	F	3.03	Pediatric	Cerebellum	Infratentorial	NA	KIAA1549 EXON 16-BRAF EXON 11	NA	NA	WT	TT	NA	NA	NA	NA	NA	21	1	1
**PA149**	F	1	Pediatric	3rd Ventricle	Supratentorial	NONE	KIAA1549 EXON 16-BRAF EXON 11	NA	NA	WT	TG	NA	Y	This study	SNP: Illumina Omni1-Quad	This study	35	1	0
**PA150**	M	2	Pediatric	Posterior fossa	Infratentorial	NONE	KIAA1549 EXON 16-BRAF EXON 9	NA	NA	WT	TT	Y	Y	This study	SNP: Illumina Omni1-Quad	This study	82	0	1
**PA151**	M	0.33	Pediatric	Occipital Lobe	Supratentorial	NONE	N	WT	WT	WT	TG	NA	Y	This study	SNP: Illumina Omni1-Quad	This study	55	0	0
**PA152**	F	4.48	Pediatric	Cerebellum	Infratentorial	NA	N	NA	NA	V600E	GG	Y	NA	NA	NA	NA	2	1	0
**PA153**	F	16.64	Pediatric	Cerebellum	Infratentorial	NONE	KIAA1549 EXON 16-BRAF EXON 9	NA	NA	WT	TT	NA	Y	This study	SNP: Illumina Omni1-Quad	This study	42	0	1
**PA154**	F	4.06	Pediatric	Cerebellum	Infratentorial	NA	KIAA1549 EXON 16-BRAF EXON 9	NA	NA	WT	TG	NA	Y	This study	NA	NA	25	0	1
**PA155**	M	11.58	Pediatric	Cerebellum	Infratentorial	NA	KIAA1549 EXON 16-BRAF EXON 9	NA	NA	WT	TT	NA	NA	NA	NA	NA	26	0	1
**PA156**	M	0.91	Pediatric	Suprasellar	Supratentorial	NONE	KIAA1549-BRAF	NA	NA	NA	TG	NA	Y	This study	SNP: Illumina Omni1-Quad	This study	0	1	0
**PA157**	F	14	Pediatric	Intraventricular	Supratentorial	NA		WT	WT	WT	TT	NA	NA	NA	NA	NA	NA	NA	NA
**PA158**	M	12	Pediatric	Thalamus	Supratentorial	NA		WT	WT	WT	TT	NA	NA	NA	NA	NA	NA	NA	NA
**PA159**	M	13	Pediatric	Spinal cord	Infratentorial	NA		WT	WT	WT	TT	NA	NA	NA	NA	NA	NA	NA	NA
**PA160**	F	13	Pediatric	Cerebellar	Infratentorial	NA		NA	NA	WT	TT	NA	NA	NA	NA	NA	NA	NA	NA
**PA161**	F	7.83333	Pediatric	Suprasellar/Optic Pathway	Supratentorial	NA	KIAA1549 EXON 16-BRAF EXON 9	WT	WT	NA	TG	NA	NA	NA	NA	NA	NA	NA	NA
**PA162**	F	38	Adult	Supratentorial	Supratentorial	Gain: +1,2	KIAA1549 EXON 16-BRAF EXON 9	NA	NA	WT	TG	NA	NA	NA	FFPE RESTORE; SNP: Illumina Omni2.5	This study	NA	NA	NA
**PA163**	M	46	Adult	Cerebellar	Infratentorial	NA	N	NA	NA	WT	TT	NA	NA	NA	NA	NA	NA	NA	NA
**PA164**	M	23	Adult	Cerebellar	Infratentorial	NA	N	NA	NA	WT	TG	NA	NA	NA	NA	NA	NA	NA	NA
**PA165**	F	63	Adult	Cerebellar	Infratentorial	NA	N	NA	NA	WT	TT	NA	NA	NA	NA	NA	NA	NA	NA
**PA166**	F	65	Adult	Cerebellar	Infratentorial	NONE	N	NA	NA	WT	TT	NA	NA	NA	FFPE RESTORE; SNP: Illumina Omni2.5	This study	NA	NA	NA
**PA167**	M	28	Adult	Spinal cord	Infratentorial	Gain: +1	KIAA1549 EXON 15-BRAF EXON 9	NA	NA	WT	TG	NA	NA	NA	FFPE RESTORE; SNP: Illumina Omni2.5	This study	NA	NA	NA
**PA168**	F	36	Adult	Supratentorial	Supratentorial	Gain: +6, 11, 13, 20, X	N	WT	NA	WT	TT	NA	NA	NA	FFPE RESTORE; SNP: Illumina Omni2.5	This study	NA	NA	NA
**PA169**	M	21	Adult	Cerebellar	Infratentorial	NA	N	NA	NA	WT	TG	NA	NA	NA	NA	NA	NA	NA	NA
**PA170**	F	40	Adult	Supratentorial	Supratentorial	NONE	N	p.K656M, p.T658P	NA	WT	TG	NA	NA	NA	FFPE RESTORE; SNP: Illumina Omni2.5	This study	122	0	NA
**PA171**	F	48	Adult	Spinal cord	Infratentorial	NA	N	NA	NA	WT	TG	NA	NA	NA	NA	NA	56	0	NA
**PA172**	F	24	Adult	Supratentorial	Supratentorial	NA	N	WT	NA	WT	TT	NA	NA	NA	NA	NA	58	0	NA
**PA173**	M	23	Adult	Cerebellar	Infratentorial	NA	N	NA	NA	WT	NA	NA	NA	NA	NA	NA	NA	NA	NA
**PA174**	M	33	Adult	Cerebellar	Infratentorial	Gain: +4,5,6,8,12,13,14,17,18,20	N	NA	NA	WT	TG	NA	NA	NA	FFPE RESTORE; SNP: Illumina Omni2.5	This study	NA	NA	NA
**PA175**	M	24	Adult	Cerebellar	Infratentorial	NA	KIAA1549 EXON 16-BRAF EXON 11	NA	NA	WT	TG	NA	NA	NA	NA	NA	NA	NA	NA
**PA176**	M	60	Adult	Spinal cord	Infratentorial	NA	KIAA1549 EXON 16-BRAF EXON 9	NA	NA	WT	TT	NA	NA	NA	NA	NA	NA	NA	NA
**PA177**	M	42	Adult	Cerebellar	Infratentorial	NONE	N	NA	NA	WT	TG	NA	NA	NA	FFPE RESTORE; SNP: Illumina Omni2.5	This study	NA	NA	NA
**PA178**	F	27	Adult	Supratentorial	Supratentorial	Gain: +1, 2,3, 4, 8, 12	KIAA1549 EXON 16-BRAF EXON 9	NA	NA	WT	TT	NA	NA	NA	FFPE RESTORE; SNP: Illumina Omni2.5	This study	NA	NA	NA
**PA179**	F	65	Adult	Supratentorial	Supratentorial	Gain: +5,6,7,9,10,11,X	N	WT	NA	WT	TT	NA	NA	NA	FFPE RESTORE; SNP: Illumina Omni2.5	This study	NA	NA	NA
**PA180**	M	38	Adult	Supratentorial	Supratentorial	NA	KIAA1549 EXON 15-BRAF EXON 9	NA	NA	WT	TG	NA	NA	NA	NA	NA	NA	NA	NA
**PA181**	F	63	Adult	Supratentorial	Supratentorial	NONE	N	p.K656M, p.T658P	NA	WT	TT	NA	NA	NA	FFPE RESTORE; SNP: Illumina Omni2.5	This study	15	0	NA
**PA182**	M	22	Adult	Cerebellar	Infratentorial	NA	KIAA1549 EXON 16-BRAF EXON 9	NA	NA	WT	TG	NA	NA	NA	NA	NA	NA	NA	NA
**PA183**	F	32	Adult	Cerebellar	Infratentorial	NA	N	NA	NA	WT	TT	NA	NA	NA	NA	NA	NA	NA	NA
**PA184**	M	44	Adult	Cerebellar	Infratentorial	NONE	N	NA	NA	WT	TT	NA	NA	NA	FFPE RESTORE; SNP: Illumina Omni2.5	This study	NA	NA	NA
**PA185**	F	34	Adult	Supratentorial	Supratentorial	NONE	N	NA	NA	WT	TG	NA	NA	NA	FFPE RESTORE; SNP: Illumina Omni2.5	This study	NA	NA	NA
**PA186**	F	23	Adult	Cerebellar	Infratentorial	NONE	N	NA	NA	WT	TG	NA	NA	NA	FFPE RESTORE; SNP: Illumina Omni2.5	This study	15	0	NA
**PA187**	F	3	Pediatric	Cerebellum	Infratentorial	NONE	KIAA1549-BRAF	NA	NA	NA	NA	NA	NA	NA	Methyl-CNV: Illumina 450K Methylation Array	Lambert et al, Acta Neuropathol 2013	33	0	1
**PA188**	F	3	Pediatric	Cerebellum	Infratentorial	NONE	KIAA1549-BRAF	WT	WT	WT	TG	NA	Y	Jones et al, Nat Genet 2013	Methyl-CNV: Illumina 450K Methylation Array	Lambert et al, Acta Neuropathol 2013	28	0	1
**PA189**	M	1	Pediatric	Optic chiasm	Supratentorial	NONE	KIAA1549-BRAF	WT	WT	WT	TG	NA	Y	Jones et al, Nat Genet 2013	Methyl-CNV: Illumina 450K Methylation Array	Lambert et al, Acta Neuropathol 2013	22	0	0
**PA190**	F	4	Pediatric	Optic tract	Supratentorial	NONE	N	WT	WT	WT	TT	NA	Y	Jones et al, Nat Genet 2013	Methyl-CNV: Illumina 450K Methylation Array	Lambert et al, Acta Neuropathol 2013	11	1	0
**PA191**	F	7	Pediatric	Cerebellum	Infratentorial	NONE	KIAA1549-BRAF	WT	WT	WT	NA	NA	Y	Jones et al, Nat Genet 2013	Methyl-CNV: Illumina 450K Methylation Array	Lambert et al, Acta Neuropathol 2013	18	0	1
**PA192**	F	15	Pediatric	4th ventricle	Infratentorial	NONE	KIAA1549-BRAF	WT	WT	WT	TT	NA	Y	Jones et al, Nat Genet 2013	Methyl-CNV: Illumina 450K Methylation Array	Lambert et al, Acta Neuropathol 2013	23	0	0
**PA193**	F	3	Pediatric	Cerebellum	Infratentorial	NONE	KIAA1549-BRAF	WT	WT	WT	TG	NA	Y	Jones et al, Nat Genet 2013	Methyl-CNV: Illumina 450K Methylation Array	Lambert et al, Acta Neuropathol 2013	14	0	1
**PA194**	F	11	Pediatric	Cerebellum	Infratentorial	+11	KIAA1549-BRAF	WT	WT	WT	TT	NA	Y	Jones et al, Nat Genet 2013	Methyl-CNV: Illumina 450K Methylation Array	Lambert et al, Acta Neuropathol 2013	21	0	1
**PA195**	F	14	Pediatric	4th ventricle	Infratentorial	+5 +6 +7 +11 +12 +16	KIAA1549-BRAF	WT	WT	WT	TG	NA	NA	NA	Methyl-CNV: Illumina 450K Methylation Array	Lambert et al, Acta Neuropathol 2013	25	0	1
**PA196**	F	5	Pediatric	3rd ventricle	Supratentorial	NONE	KIAA1549-BRAF	WT	WT	WT	GG	NA	Y	Jones et al, Nat Genet 2013	Methyl-CNV: Illumina 450K Methylation Array	Lambert et al, Acta Neuropathol 2013	23	0	0
**PA197**	M	4	Pediatric	Brainstem	Infratentorial	NONE	KIAA1549-BRAF	WT	WT	WT	TG	NA	Y	Jones et al, Nat Genet 2013	Methyl-CNV: Illumina 450K Methylation Array	Lambert et al, Acta Neuropathol 2013	24	0	0
**PA198**	F	23	Adult	Optic tract	Supratentorial	NONE	KIAA1549-BRAF	WT	WT	WT	TT	NA	Y	Jones et al, Nat Genet 2013	Methyl-CNV: Illumina 450K Methylation Array	Lambert et al, Acta Neuropathol 2013	23	0	1
**PA199**	F	15	Pediatric	Optic chiasm	Supratentorial	NONE	KIAA1549-BRAF	WT	WT	WT	GG	NA	Y	Jones et al, Nat Genet 2013	Methyl-CNV: Illumina 450K Methylation Array	Lambert et al, Acta Neuropathol 2013	24	0	0
**PA200**	M	4	Pediatric	Cerebellum	Infratentorial	+5 +6 ++8 +10 +11 +15 +18	KIAA1549-BRAF	WT	WT	WT	TG	NA	Y	Jones et al, Nat Genet 2013	Methyl-CNV: Illumina 450K Methylation Array	Lambert et al, Acta Neuropathol 2013	22	0	1
**PA201**	F	1	Pediatric	Cerebellum	Infratentorial	NONE	NA	NA	NA	NA	NA	NA	NA	NA	Methyl-CNV: Illumina 450K Methylation Array	Lambert et al, Acta Neuropathol 2013	26	0	1
**PA202**	M	2	Pediatric	Optic chiasm	Supratentorial	NONE	KIAA1549-BRAF	WT	WT	WT	GG	NA	Y	Jones et al, Nat Genet 2013	Methyl-CNV: Illumina 450K Methylation Array	Lambert et al, Acta Neuropathol 2013	21	0	1
**PA203**	M	3	Pediatric	4th ventricle	Infratentorial	NONE	KIAA1549-BRAF	WT	WT	WT	TG	NA	NA	NA	Methyl-CNV: Illumina 450K Methylation Array	Lambert et al, Acta Neuropathol 2013	24	0	1
**PA204**	F	15	Pediatric	Brainstem	Infratentorial	NONE	N	p.K655I, p.K656E	WT	WT	TT	NA	Y	Jones et al, Nat Genet 2013	Methyl-CNV: Illumina 450K Methylation Array	Lambert et al, Acta Neuropathol 2013	NA	NA	0
**PA205**	M	3	Pediatric	Cerebellum	Infratentorial	NONE	KIAA1549-BRAF	WT	WT	WT	TG	NA	Y	Jones et al, Nat Genet 2013	Methyl-CNV: Illumina 450K Methylation Array	Lambert et al, Acta Neuropathol 2013	24	0	1
**PA206**	F	6	Pediatric	Brainstem	Infratentorial	NONE	KIAA1549-BRAF	WT	WT	WT	TG	NA	Y	Jones et al, Nat Genet 2013	Methyl-CNV: Illumina 450K Methylation Array	Lambert et al, Acta Neuropathol 2013	10	1	0
**PA207**	M	9	Pediatric	Optic tract	Supratentorial	NONE	KIAA1549-BRAF	WT	WT	WT	TG	NA	NA	NA	Methyl-CNV: Illumina 450K Methylation Array	Lambert et al, Acta Neuropathol 2013	12	0	1
**PA208**	F	3	Pediatric	3rd ventricle	Supratentorial	NONE	KIAA1549-BRAF	WT	WT	WT	TG	NA	Y	Jones et al, Nat Genet 2013	Methyl-CNV: Illumina 450K Methylation Array	Lambert et al, Acta Neuropathol 2013	3	1	0
**PA209**	M	6	Pediatric	Brainstem	Infratentorial	NONE	KIAA1549-BRAF	WT	WT	WT	GG	NA	Y	Jones et al, Nat Genet 2013	Methyl-CNV: Illumina 450K Methylation Array	Lambert et al, Acta Neuropathol 2013	4	1	1
**PA210**	F	6	Pediatric	Cerebellum	Infratentorial	NONE	KIAA1549-BRAF	NA	NA	NA	NA	NA	NA	NA	Methyl-CNV: Illumina 450K Methylation Array	Lambert et al, Acta Neuropathol 2013	5	1	1
**PA211**	M	10	Pediatric	Cerebellum	Infratentorial	NONE	KIAA1549-BRAF	WT	WT	WT	GG	NA	Y	Jones et al, Nat Genet 2013	Methyl-CNV: Illumina 450K Methylation Array	Lambert et al, Acta Neuropathol 2013	15	0	1
**PA212**	F	4	Pediatric	Thalamus	Supratentorial	NONE	N	NA	NA	WT	NA	NA	NA	NA	Methyl-CNV: Illumina 450K Methylation Array	Lambert et al, Acta Neuropathol 2013	4	1	0
**PA213**	F	5	Pediatric	Cerebellum	Infratentorial	NONE	KIAA1549-BRAF	WT	WT	WT	GG	NA	Y	Jones et al, Nat Genet 2013	Methyl-CNV: Illumina 450K Methylation Array	Lambert et al, Acta Neuropathol 2013	4	1	0
**PA214**	M	9	Pediatric	Cerebellum	Infratentorial	NONE	KIAA1549-BRAF	WT	WT	WT	GG	NA	Y	Jones et al, Nat Genet 2013	Methyl-CNV: Illumina 450K Methylation Array	Lambert et al, Acta Neuropathol 2013	12	0	1
**PA215**	F	6	Pediatric	Cerebellum	Infratentorial	NONE	KIAA1549-BRAF	NA	NA	NA	NA	NA	NA	NA	Methyl-CNV: Illumina 450K Methylation Array	Lambert et al, Acta Neuropathol 2013	8	1	1
**PA216**	M	13	Pediatric	Brainstem	Infratentorial	+5 +6 +7 +11 +12	KIAA1549-BRAF	WT	WT	WT	TG	NA	Y	Jones et al, Nat Genet 2013	Methyl-CNV: Illumina 450K Methylation Array	Lambert et al, Acta Neuropathol 2013	12	0	1
**PA217**	F	5	Pediatric	Cerebellum	Infratentorial	NONE	KIAA1549-BRAF	WT	WT	WT	TG	NA	Y	Jones et al, Nat Genet 2013	Methyl-CNV: Illumina 450K Methylation Array	Lambert et al, Acta Neuropathol 2013	24	0	0
**PA218**	F	4	Pediatric	Cerebellum	Infratentorial	NONE	KIAA1549-BRAF	WT	WT	WT	NA	NA	NA	NA	Methyl-CNV: Illumina 450K Methylation Array	Lambert et al, Acta Neuropathol 2013	7	1	0
**PA219**	F	17	Pediatric	Brainstem	Infratentorial	+5 +6 +7 +11	KIAA1549-BRAF	WT	WT	WT	TG	NA	Y	Jones et al, Nat Genet 2013	Methyl-CNV: Illumina 450K Methylation Array	Lambert et al, Acta Neuropathol 2013	12	0	1
**PA220**	M	4	Pediatric	Brainstem	Infratentorial	NONE	KIAA1549-BRAF	WT	WT	WT	TG	NA	NA	NA	Methyl-CNV: Illumina 450K Methylation Array	Lambert et al, Acta Neuropathol 2013	20	0	0
**PA221**	M	16	Pediatric	Optic tract	Supratentorial	+5 +6	N	WT	WT	p.R506_insVLR	TG	NA	Y	Jones et al, Nat Genet 2013	Methyl-CNV: Illumina 450K Methylation Array	Lambert et al, Acta Neuropathol 2013	10	0	1
**PA222**	F	6	Pediatric	Thalamus	Supratentorial	NONE	N	p.N546K	WT	WT	TT	NA	Y	Jones et al, Nat Genet 2013	Methyl-CNV: Illumina 450K Methylation Array	Lambert et al, Acta Neuropathol 2013	17	1	0
**PA223**	M	6	Pediatric	Cerebellum	Infratentorial	NONE	KIAA1549-BRAF	WT	WT	WT	TG	NA	Y	Jones et al, Nat Genet 2013	Methyl-CNV: Illumina 450K Methylation Array	Lambert et al, Acta Neuropathol 2013	12	0	1
**PA224**	F	2	Pediatric	Optic nerve	Supratentorial	NONE	N	WT	WT	WT	TT	NA	NA	NA	Methyl-CNV: Illumina 450K Methylation Array	Lambert et al, Acta Neuropathol 2013	5	1	0
**PA225**	M	9	Pediatric	Cerebellum	Infratentorial	+5 +6 +11 +15 +20 +21	KIAA1549-BRAF	WT	WT	WT	TG	NA	NA	NA	Methyl-CNV: Illumina 450K Methylation Array	Lambert et al, Acta Neuropathol 2013	10	0	1
**PA226**	M	14	Pediatric	Optic tract	Supratentorial	+5 +6 +7 +10 +12 +14 +18 +19 +20 +21 +22 +X	N	WT	WT	p.V600E	TT	NA	NA	NA	Methyl-CNV: Illumina 450K Methylation Array	Lambert et al, Acta Neuropathol 2013	12	0	1
**PA227**	M	6	Pediatric	Cerebellum	Infratentorial	NONE	KIAA1549-BRAF	WT	WT	WT	TG	NA	Y	Jones et al, Nat Genet 2013	Methyl-CNV: Illumina 450K Methylation Array	Lambert et al, Acta Neuropathol 2013	8	0	1
**PA228**	M	14	Pediatric	Cerebellum	Infratentorial	NONE	KIAA1549-BRAF	WT	WT	WT	TG	NA	Y	Jones et al, Nat Genet 2013	Methyl-CNV: Illumina 450K Methylation Array	Lambert et al, Acta Neuropathol 2013	10	0	1
**PA229**	M	12	Pediatric	Optic tract	Supratentorial	NONE	N	WT	WT	WT	GG	NA	Y	Jones et al, Nat Genet 2013	Methyl-CNV: Illumina 450K Methylation Array	Lambert et al, Acta Neuropathol 2013	7	0	NA
**PA230**	F	8	Pediatric	Brainstem	Infratentorial	NONE	KIAA1549-BRAF	WT	WT	WT	TG	NA	Y	Jones et al, Nat Genet 2013	Methyl-CNV: Illumina 450K Methylation Array	Lambert et al, Acta Neuropathol 2013	12	0	1
**PA231**	M	9	Pediatric	3rd ventricle	Supratentorial	NONE	N	p.K656E	p.E76A	WT	GG	NA	NA	NA	Methyl-CNV: Illumina 450K Methylation Array	Lambert et al, Acta Neuropathol 2013	10	0	0
**PA232**	F	4	Pediatric	Cerebellum	Infratentorial	NONE	KIAA1549-BRAF	WT	WT	WT	TT	NA	Y	Jones et al, Nat Genet 2013	Methyl-CNV: Illumina 450K Methylation Array	Lambert et al, Acta Neuropathol 2013	7	0	1
**PA233**	M	11	Pediatric	Cerebellum	Infratentorial	NONE	KIAA1549-BRAF	WT	WT	WT	TT	NA	Y	Jones et al, Nat Genet 2013	Methyl-CNV: Illumina 450K Methylation Array	Lambert et al, Acta Neuropathol 2013	8	0	1
**PA234**	F	4	Pediatric	Cerebellum	Infratentorial	NONE	KIAA1549-BRAF	WT	WT	WT	TG	NA	Y	Jones et al, Nat Genet 2013	Methyl-CNV: Illumina 450K Methylation Array	Lambert et al, Acta Neuropathol 2013	10	0	1
**PA235**	F	6	Pediatric	Cerebellum	Infratentorial	NONE	KIAA1549-BRAF	WT	WT	WT	GG	NA	Y	Jones et al, Nat Genet 2013	Methyl-CNV: Illumina 450K Methylation Array	Lambert et al, Acta Neuropathol 2013	8	0	1
**PA236**	M	8	Pediatric	Brainstem	Infratentorial	NONE	N	ITD Kinase Domain	WT	WT	GG	NA	Y	Jones et al, Nat Genet 2013	Methyl-CNV: Illumina 450K Methylation Array	Lambert et al, Acta Neuropathol 2013	6	0	0
**PA237**	M	7	Pediatric	Frontal lobe	Supratentorial	NONE	N	NA	NA	WT	NA	NA	NA	NA	Methyl-CNV: Illumina 450K Methylation Array	Lambert et al, Acta Neuropathol 2013	11	0	1
**PA238**	F	3	Pediatric	Brainstem	Infratentorial	NONE	KIAA1549-BRAF	WT	WT	WT	TG	NA	Y	Jones et al, Nat Genet 2013	Methyl-CNV: Illumina 450K Methylation Array	Lambert et al, Acta Neuropathol 2013	6	0	0
**PA239**	M	12	Pediatric	4th ventricle	Infratentorial	+4 +5 +6 +8 +11 +12	N	p.N546K	WT	WT	TT	NA	Y	Jones et al, Nat Genet 2013	Methyl-CNV: Illumina 450K Methylation Array	Lambert et al, Acta Neuropathol 2013	4	0	1
**PA240**	F	21	Adult	Cerebellum	Infratentorial	NONE	KIAA1549-BRAF	WT	WT	WT	TG	NA	Y	Jones et al, Nat Genet 2013	Methyl-CNV: Illumina 450K Methylation Array	Lambert et al, Acta Neuropathol 2013	7	0	1
**PA241**	F	4	Pediatric	Cerebellum	Infratentorial	NONE	KIAA1549-BRAF	WT	WT	WT	TG	NA	Y	Jones et al, Nat Genet 2013	Methyl-CNV: Illumina 450K Methylation Array	Lambert et al, Acta Neuropathol 2013	14	1	1
**PA242**	F	7	Pediatric	Cerebellum	Infratentorial	NONE	KIAA1549-BRAF	WT	WT	WT	TG	NA	Y	Jones et al, Nat Genet 2013	Methyl-CNV: Illumina 450K Methylation Array	Lambert et al, Acta Neuropathol 2013	8	0	1
**PA243**	F	15	Pediatric	Brainstem	Infratentorial	NONE	KIAA1549-BRAF	WT	WT	WT	TG	NA	Y	Jones et al, Nat Genet 2013	Methyl-CNV: Illumina 450K Methylation Array	Lambert et al, Acta Neuropathol 2013	7	0	1
**PA244**	F	8	Pediatric	Cerebellum	Infratentorial	NONE	KIAA1549-BRAF	WT	WT	WT	TG	NA	Y	Jones et al, Nat Genet 2013	Methyl-CNV: Illumina 450K Methylation Array	Lambert et al, Acta Neuropathol 2013	12	0	1
**PA245**	M	9	Pediatric	Cerebellum	Infratentorial	NONE	KIAA1549-BRAF	WT	WT	WT	NA	NA	Y	Jones et al, Nat Genet 2013	Methyl-CNV: Illumina 450K Methylation Array	Lambert et al, Acta Neuropathol 2013	7	0	1
**PA246**	F	1	Pediatric	Cerebellum	Infratentorial	NONE	KIAA1549-BRAF	WT	WT	WT	TG	NA	Y	Jones et al, Nat Genet 2013	Methyl-CNV: Illumina 450K Methylation Array	Lambert et al, Acta Neuropathol 2013	11	0	1
**PA247**	M	15	Pediatric	Cerebellum	Infratentorial	NONE	KIAA1549-BRAF	WT	WT	WT	TT	NA	Y	Jones et al, Nat Genet 2013	Methyl-CNV: Illumina 450K Methylation Array	Lambert et al, Acta Neuropathol 2013	4	0	1
**PA248**	M	7	Pediatric	3rd ventricle	Supratentorial	NONE	KIAA1549-BRAF	WT	WT	WT	TG	NA	Y	Jones et al, Nat Genet 2013	Methyl-CNV: Illumina 450K Methylation Array	Lambert et al, Acta Neuropathol 2013	8	0	1
**PA249**	F	13	Pediatric	Cerebellum	Infratentorial	NONE	KIAA1549-BRAF	WT	WT	WT	TG	NA	Y	Jones et al, Nat Genet 2013	Methyl-CNV: Illumina 450K Methylation Array	Lambert et al, Acta Neuropathol 2013	4	0	1
**PA250**	F	4	Pediatric	3rd ventricle	Supratentorial	NONE	KIAA1549-BRAF	WT	WT	WT	TT	NA	Y	Jones et al, Nat Genet 2013	Methyl-CNV: Illumina 450K Methylation Array	Lambert et al, Acta Neuropathol 2013	8	0	1
**PA251**	F	5	Pediatric	Brainstem	Infratentorial	NONE	KIAA1549-BRAF	WT	WT	WT	TG	NA	Y	Jones et al, Nat Genet 2013	Methyl-CNV: Illumina 450K Methylation Array	Lambert et al, Acta Neuropathol 2013	4	0	0
**PA252**	F	5	Pediatric	Cerebellum	Infratentorial	NONE	KIAA1549-BRAF	WT	WT	WT	TT	NA	Y	Jones et al, Nat Genet 2013	Methyl-CNV: Illumina 450K Methylation Array	Lambert et al, Acta Neuropathol 2013	9	0	1
**PA253**	M	12	Pediatric	Cerebellum	Infratentorial	NONE	KIAA1549-BRAF	WT	WT	WT	TT	NA	Y	Jones et al, Nat Genet 2013	Methyl-CNV: Illumina 450K Methylation Array	Lambert et al, Acta Neuropathol 2013	3	0	1
**PA254**	M	14	Pediatric	Parietal lobe	Supratentorial	NONE	KIAA1549-BRAF	WT	WT	WT	TT	NA	Y	Jones et al, Nat Genet 2013	Methyl-CNV: Illumina 450K Methylation Array	Lambert et al, Acta Neuropathol 2013	8	0	1
**PA255**	F	50	Adult	Cerebellum	Infratentorial	+5 +6 +10 +11	KIAA1549-BRAF	WT	WT	WT	GG	NA	Y	Jones et al, Nat Genet 2013	Methyl-CNV: Illumina 450K Methylation Array	Lambert et al, Acta Neuropathol 2013	6	0	1
**PA256**	F	40	Adult	4th ventricle	Infratentorial	NONE	N	WT	WT	WT	TT	NA	Y	Jones et al, Nat Genet 2013	Methyl-CNV: Illumina 450K Methylation Array	Lambert et al, Acta Neuropathol 2013	4	0	0
**PA257**	F	7	Pediatric	Optic nerve	Supratentorial	+4 +5 ++6 ++7 +8 +10 +11	KIAA1549-BRAF	NA	NA	NA	NA	NA	NA	NA	Methyl-CNV: Illumina 450K Methylation Array	Lambert et al, Acta Neuropathol 2013	NA	NA	0
**PA258**	F	9	Pediatric	4th ventricle	Infratentorial	NONE	KIAA1549-BRAF	WT	WT	WT	GG	NA	NA	NA	Methyl-CNV: Illumina 450K Methylation Array	Lambert et al, Acta Neuropathol 2013	46	0	1
**PA259**	M	7	Pediatric	Brainstem	Infratentorial	NONE	N	WT	WT	WT	TG	NA	NA	NA	Methyl-CNV: Illumina 450K Methylation Array	Lambert et al, Acta Neuropathol 2013	6	0	0
**PA260**	M	3	Pediatric	Cerebellum	Infratentorial	NONE	KIAA1549-BRAF	WT	WT	WT	GG	NA	NA	NA	Methyl-CNV: Illumina 450K Methylation Array	Lambert et al, Acta Neuropathol 2013	7	0	1
**PA261**	F	14	Pediatric	Cerebellum	Infratentorial	NONE	KIAA1549-BRAF	WT	WT	WT	TG	NA	NA	NA	Methyl-CNV: Illumina 450K Methylation Array	Lambert et al, Acta Neuropathol 2013	4	0	NA
**PA262**	F	11	Pediatric	Cerebellum	Infratentorial	NONE	KIAA1549-BRAF	WT	WT	WT	TG	NA	NA	NA	Methyl-CNV: Illumina 450K Methylation Array	Lambert et al, Acta Neuropathol 2013	4	0	1
**PA263**	F	10	Pediatric	3rd ventricle	Supratentorial	4 +5 +6 +7 +8 +9 +10 +11 +12 +16 +20 +21	N	WT	WT	p.T599_insT	TG	NA	NA	NA	Methyl-CNV: Illumina 450K Methylation Array	Lambert et al, Acta Neuropathol 2013	4	0	1
**PA264**	F	22	Adult	Cerebellum	Infratentorial	NONE	N	WT	WT	WT	TG	NA	NA	NA	Methyl-CNV: Illumina 450K Methylation Array	Lambert et al, Acta Neuropathol 2013	4	0	1
**PA265**	F	17	Pediatric	4th ventricle	Infratentorial	+5 +6 +7 +10 +X	N	WT	WT	WT	TG	NA	NA	NA	Methyl-CNV: Illumina 450K Methylation Array	Lambert et al, Acta Neuropathol 2013	5	0	0
**PA266**	F	2	Pediatric	Optic tract	Supratentorial	NONE	KIAA1549-BRAF	WT	WT	WT	TG	NA	NA	NA	Methyl-CNV: Illumina 450K Methylation Array	Lambert et al, Acta Neuropathol 2013	4	0	0
**PA267**	F	7	Pediatric	Cerebellum	Infratentorial	NONE	KIAA1549-BRAF	WT	WT	WT	TT	NA	NA	NA	Methyl-CNV: Illumina 450K Methylation Array	Lambert et al, Acta Neuropathol 2013	4	0	1
**PA268**	M	12	Pediatric	Cerebellum	Infratentorial	NONE	KIAA1549-BRAF	WT	WT	WT	TT	NA	NA	NA	Methyl-CNV: Illumina 450K Methylation Array	Lambert et al, Acta Neuropathol 2013	5	0	0
**PA269**	M	1	Pediatric	3rd ventricle	Supratentorial	NONE	KIAA1549-BRAF	WT	WT	WT	GG	NA	NA	NA	Methyl-CNV: Illumina 450K Methylation Array	Lambert et al, Acta Neuropathol 2013	3	1	0
**PA270**	M	2	Pediatric	Cerebellum	Infratentorial	NONE	KIAA1549-BRAF	WT	WT	WT	TT	NA	NA	NA	Methyl-CNV: Illumina 450K Methylation Array	Lambert et al, Acta Neuropathol 2013	5	0	1
**PA271**	F	11	Pediatric	Cerebellum	Infratentorial	NONE	KIAA1549-BRAF	WT	WT	WT	TG	NA	NA	NA	Methyl-CNV: Illumina 450K Methylation Array	Lambert et al, Acta Neuropathol 2013	3	0	1
**PA272**	F	5	Pediatric	Brainstem	Infratentorial	NONE	N	WT	WT	p.V600E	TG	NA	NA	NA	Methyl-CNV: Illumina 450K Methylation Array	Lambert et al, Acta Neuropathol 2013	3	0	0
**PA273**	M	5	Pediatric	Optic tract	Supratentorial	NONE	N	WT	WT	WT	TT	NA	NA	NA	Methyl-CNV: Illumina 450K Methylation Array	Lambert et al, Acta Neuropathol 2013	3	0	0
**PA274**	M	1	Pediatric	Cerebellum	Infratentorial	NONE	KIAA1549-BRAF	WT	WT	WT	TG	NA	NA	NA	Methyl-CNV: Illumina 450K Methylation Array	Lambert et al, Acta Neuropathol 2013	3	0	1
**PA275**	M	7	Pediatric	Cerebellum	Infratentorial	NONE	KIAA1549-BRAF	WT	WT	WT	NA	NA	NA	NA	Methyl-CNV: Illumina 450K Methylation Array	Lambert et al, Acta Neuropathol 2013	3	0	1
**PA276**	F	29	Adult	Brainstem	Infratentorial	NONE	N	WT	WT	p.V600E	TG	NA	NA	NA	Methyl-CNV: Illumina 450K Methylation Array	Lambert et al, Acta Neuropathol 2013	3	0	0
**PA277**	M	5	Pediatric	Brainstem	Infratentorial	+5 +6	KIAA1549-BRAF	WT	WT	WT	GG	NA	NA	NA	Methyl-CNV: Illumina 450K Methylation Array	Lambert et al, Acta Neuropathol 2013	3	0	0

**PA278**	F	8	Pediatric	Hypothalamus	Supratentorial	NONE	N	p.N546K	p.E69K	WT	TG	NA	Y	Jones et al, Nat Genet 2013	Methyl-CNV: Illumina 450K Methylation Array	Lambert et al, Acta Neuropathol 2013	4	1	0
**PA279**	F	2	Pediatric	Diencephalon	Supratentorial	NONE	KIAA1549-BRAF	WT	WT	WT	NA	NA	Y	Jones et al, Nat Genet 2013	aCGH	Cin et al, Acta Neuropathol 2011	22	1	0
**PA280**	M	3	Pediatric	Diencephalon	Supratentorial	NONE	KIAA1549-BRAF	WT	WT	WT	NA	NA	Y	Jones et al, Nat Genet 2013	aCGH	Cin et al, Acta Neuropathol 2011	12	1	0
