# Supplementary material for: Non-random aneuploidy specifies subgroups of pilocytic astrocytoma and correlates with older age
Source: Oncotarget. 2015 Sep 10;6(31):31844–56. doi: 10.18632/oncotarget.5571 (PMC4741644; doi:10.18632/oncotarget.5571)
Supplement: Supplementary file 3 [file oncotarget-06-31844-s003.pdf]

**Supplementary Table 2: Gene Ontology Analysis of 741 MDM2-correlated genes in 122 pilocytic astrocytoma samples with available aneuploid status at FDR<0.001**

| R# | # | _p_value_ | Goid-Desc | GeneSymbols |
|---|---|---|---|---|
| 11 | 5 | 4.60E-09 | 4861: cyclin-dependent protein kinase inhibitor activity | CDKN1A, CDKN1B, CDKN2A, CDKN2B, CDKN2C |
| 50 | 9 | 1.70E-05 | 79: regulation of cyclin-dependent protein kinase activity | CDKN1A, CDKN1B, CDKN2B, CDKN2C, GTF2H1, CCNL1, CCNL2, CCNT2, CCNE2 |
| 50 | 9 | 1.70E-05 | 79: regulation of cyclin-dependent protein kinase activity | CDKN1A, CDKN1B, CDKN2B, CDKN2C, GTF2H1, CCNL1, CCNL2, CCNT2, CCNE2 |
| 18 | 5 | 2.40E-05 | 42326: negative regulation of phosphorylation | CDKN1A, CDKN1B, CDKN2A, CDKN2B, CDKN2C |
| 18 | 5 | 2.40E-05 | 42326: negative regulation of phosphorylation | CDKN1A, CDKN1B, CDKN2A, CDKN2B, CDKN2C |
| 18 | 5 | 2.40E-05 | 51537: 2 iron, 2 sulfur cluster binding | AOX1, NDUFS1, GLRX5, CISD1, CMAHP |
| 21 | 5 | 1.60E-04 | 15078: hydrogen ion transporter activity | TCIRG1, ATP5S, ATP6V0A4, ATP5G3, ATP5J |
| 61 | 9 | 3.30E-04 | 6396: RNA processing | RBM6, DDX17, ZFC3H1, CCNL1, RBMS2, BICD1, PABPN1, CCNL2, RBM39 |
| 57 | 8 | 1.40E-03 | 51540: metal cluster binding | AOX1, NDUFS1, NDUFS2, GLRX5, CISD1, NUBPL, ISCA1, CMAHP |
| 46 | 7 | 1.50E-03 | 8037: cell recognition | DYNLL2, VCAN, OPCML, AMIGO1, ROBO3, CLEC7A, CNTNAP3 |
| 36 | 6 | 1.60E-03 | 48839: inner ear development | CDKN1B, PDGFRA, SHH, BMP2, CXCL14, LPPR4 |
| 36 | 6 | 1.60E-03 | 48839: inner ear development | CDKN1B, PDGFRA, SHH, BMP2, CXCL14, LPPR4 |
| 71 | 9 | 2.10E-03 | 7409: axonogenesis | SLITRK1, CNP, SLITRK4, NTNG1, SLITRK3, SLITRK5, ATL1, SNAP25, LPPR4 |
| 125 | 13 | 2.60E-03 | 75: cell cycle checkpoint | CDKN1A, CDKN1B, CDKN2A, PSME4, ANAPC4, MAD2L1, MDM2, ATM, ANAPC7, ATR, PSMB6, XPC, CCNE2 |
| 444 | 33 | 3.00E-03 | 16874: ligase activity | TRIM22, RNF13, HECTD2, RNF187, DDB2, UBR4, HERC4, GART, FBXO2, ANAPC4, HARS, MARS, MDM2, UBR5, UBE2R2, NADSYN1, CHFR, ACSS2, BIRC6, RIMKLB, RNF213, CCNB1IP1, MCCC2, TRAF5, UBE2E2, UBE2V2, ACSF2, TARS2, LNX1, SUCLG1, TRIM41, TTLL4, RNF144A |
| 2109 | 120 | 3.70E-03 | 166: nucleotide binding | over 100 entries (50, 70, ) |
| 31 | 5 | 6.60E-03 | 42552: myelination | OLIG2, ERBB2, NAB2, AMIGO1, GAL3ST1 |
| 860 | 54 | 9.90E-03 | 42995: cell projection | FZD10, EFHC1, PLK3, APOA1BP, DSCAM, EPHA5, ERBB2, PTK2B, ENKUR, FGF13, PALLD, SRGAP2, CADM2, ATXN10, FBXO2, AMPH, GLI3, GPM6A, AQP11, GRIA4, GRM7, LRRTM1, KCND2, MSN, MYO7A, NOS1, ATP6V0A4, ATL1, SSH1, AHI1, ENAH, SEPT11, AMIGO1, KLHL1, RAC3, RASA1, CLIP1, ROBO3, PCDH15, SNAP25, VAMP1, TNFRSF1A, FZD4, ARHGAP24, CASP8, URI1, IQGAP1, WASF1, BOC, DNER, LPXN, CRIPT, TTLL4, DOCK4 |
| 98 | 10 | 0.01 | 7417: central nervous system development | DLL3, MOG, NGFR, AHI1, SHH, SOX3, TIMP4, UGT8, ADAM23, DNER |
| 98 | 10 | 0.01 | 7417: central nervous system development | DLL3, MOG, NGFR, AHI1, SHH, SOX3, TIMP4, UGT8, ADAM23, DNER |
| 127 | 12 | 0.01 | 7050: cell cycle arrest | CDKN1A, CDKN1B, CDKN2A, CDKN2B, CDKN2C, DBC1, MACF1, GAS6, ATM, RPRM, TBRG1, TP53INP1 |
| 45 | 6 | 0.01 | 19903: protein phosphatase binding | CDKN1B, ERBB2, PPP1R16B, ANAPC4, ANAPC7, IQGAP1 |
| 47 | 6 | 0.02 | 42787: protein ubiquitination during ubiquitin-dependent protein catabolic process | HECTD2, HERC4, RNF11, MDM2, UBR5, LNX1 |
| 35 | 5 | 0.02 | 48583: regulation of response to stimulus | CDKN2A, MDM2, ATM, OMG, ATR |
| 63 | 7 | 0.02 | 6977: DNA damage response, signal transduction by p53 class mediator resulting in cell cycle arrest | CDKN1A, CDKN1B, PSME4, MDM2, ATM, TRIAP1, PSMB6 |
| 63 | 7 | 0.02 | 6977: DNA damage response, signal transduction by p53 class mediator resulting in cell cycle arrest | CDKN1A, CDKN1B, PSME4, MDM2, ATM, TRIAP1, PSMB6 |
| 37 | 5 | 0.02 | 80: G1 phase of mitotic cell cycle | CDKN1A, CDKN1B, CDKN2A, CDKN2B, CDKN2C |
| 37 | 5 | 0.02 | 80: G1 phase of mitotic cell cycle | CDKN1A, CDKN1B, CDKN2A, CDKN2B, CDKN2C |
| 37 | 5 | 0.02 | 1948: glycoprotein binding | CNTN1, ERBB2, FBXO2, RASA1, SHH |
| 36 | 5 | 0.02 | 9898: internal side of plasma membrane | G6PD, IKBKB, MSN, PTPN4, TRAF5 |
| 39 | 5 | 0.03 | 51291: protein heterooligomerization | GLRB, KCND2, SEPT11, CASP8, PCBD2 |
| 155 | 13 | 0.03 | 5516: calmodulin binding | ENKUR, UBR4, MYO1F, MYO7A, NOS1, PHKA1, PPP3CA, PPP3R1, CDK5RAP2, RIT2, MARCKSL1, CASK, IQGAP1 |
| 156 | 13 | 0.03 | 30424: axon | DSCAM, EPHA5, PTK2B, FGF13, CADM2, GRM7, LRRTM1, ATL1, SEPT11, AMIGO1, ROBO3, TNFRSF1A, BOC |
| 202 | 16 | 0.03 | 44463: cell projection part | EFHC1, SPATA18, C2CD3, FBXO2, AMPH, GPM6A, GRIA4, GRM7, KCND2, NOS1, AHI1, SEPT11, DNALI1, CEP290, CRIPT, TTLL4 |
| 81 | 8 | 0.04 | 45471: response to ethanol | PTK2B, HSD17B3, ATP5G3, SHH, TNFRSF1A, TRH, CASP8, GGH |
| 97 | 9 | 0.04 | 5741: mitochondrial outer membrane | SPATA18, MLXIP, SLC44A1, MARC2, CISD1, VAMP1, MYO19, CASP8, WASF1 |
| 318 | 22 | 0.04 | 30054: cell junction | GSG1L, SYT11, SRGAP2, FRRS1L, CADM2, AMPH, GLRB, GRIA4, LRRTM1, CHRNA9, FERMT1, ENAH, SEPT11, CDK5RAP2, SNAP25, VAMP1, SYN2, TMEM163, NETO1, IQGAP1, DLGAP1, CRIPT |
| 97 | 9 | 0.04 | 5741: mitochondrial outer membrane | SPATA18, MLXIP, SLC44A1, MARC2, CISD1, VAMP1, MYO19, CASP8, WASF1 |
| 1337 | 74 | 0.05 | 5524: ATP binding | CDK8, ATP8A1, DDX17, PMVK, IRAK3, SGK494, PLK3, STK35, KIF18B, ABCA13, DGKA, DHX36, EPHA5, ERBB2, PTK2B, CUL9, MDN1, PPIP5K2, PAN3, GART, AK5, TPK1, STK39, HARS, HUNK, EHD3, HSPA6, IKBKB, MAK, MARS, MAT2A, ABCC1, MYO1F, MYO7A, ATM, NME8, PDGFRA, ATR, DDX56, UBE2R2, SLFN12, NADSYN1, DDX19A, BMP2K, PRKACB, PRKCB, ACSS2, PRKG2, PAK7, CLK4, RIMKLB, STARD9, DDX55, KIF13A, MCCC2, YTHDC2, SYN2, TEP1, UBE2E2, MYO19, ACSF2, TARS2, NUBPL, NLRC5, MKNK1, CASK, RIPK1, MAP3K14, BAZ1B, BTAF1, DGKI, TTLL4, TLK1, THRAP3 |
| 42 | 5 | 0.05 | 30175: filopodium | FGF13, GPM6A, MSN, ENAH, SNAP25 |
