# Supplementary material for: Non-random aneuploidy specifies subgroups of pilocytic astrocytoma and correlates with older age
Source: Oncotarget. 2015 Sep 10;6(31):31844–56. doi: 10.18632/oncotarget.5571 (PMC4741644; doi:10.18632/oncotarget.5571)
Supplement: Supplementary file 4 [file oncotarget-06-31844-s004.pdf]

Supplementary Table 3: Characteristics of tumors with *BRAF* duplication/fusion included in the study

	***BRAF* duplication/fusion**	**No *BRAF* duplication/fusion***	***P*-value**
**Total (n)**	175	91	NA
**Supratentorial (n)**	37	44	**<0.0001**
Cerebral Hemispheres	7	14	**0.0029**
Thalamus/Diencephalon	6	8	NS
Optic Pathway	11	10	NS
Hypothalamus	2	2	NS
3rd ventricle	7	3	NS
Suprasellar/Supratentorial NOS	4	7	**0.05**
**Infratentorial (n)**	138	47	**<0.0001**
Cerebellum/Posterior Fossa	114	34	**<0.0001**
Brainstem	15	8	NS
4th ventricle	7	4	NS
Spinal cord	2	1	NS
**Aneuploidy (n)**	27	23	**0.0076**
***FGFR1* mutations (n)**	0	8	**<0.0001**
***BRAF* mutations (n)**	2	18	**<0.0001**
**Mean Age (years)**	11.1 years	19.8 years	**2x10-7**
**Median survival (months)**	226 months	195 months	NS

**Excludes tumors with known SRGAP3-RAF1 fusions*

NS=Not significant
